# A model based on meta-analysis to evaluate poor prognosis of patients with severe fever with thrombocytopenia syndrome

**DOI:** 10.3389/fmicb.2023.1307960

**Published:** 2024-01-08

**Authors:** Zishuai Liu, Zhouling Jiang, Ligang Zhang, Xiaoyu Xue, Chenxi Zhao, Yanli Xu, Wei Zhang, Ling Lin, Zhihai Chen

**Affiliations:** ^1^Department of Infectious Disease, Beijing Ditan Hospital, Capital Medical University, Beijing, China; ^2^Department of Infectious Diseases, Yantai Qishan Hospital, Yantai, China

**Keywords:** SFTS, risk factors, meta-analysis, prediction model, cohort study

## Abstract

**Background:**

Early identification of risk factors associated with poor prognosis in Severe fever with thrombocytopenia syndrome (SFTS) patients is crucial to improving patient survival.

**Method:**

Retrieve literature related to fatal risk factors in SFTS patients in the database, extract the risk factors and corresponding RRs and 95% CIs, and merge them. Statistically significant factors were included in the model, and stratified and assigned a corresponding score. Finally, a validation cohort from Yantai Qishan Hospital in 2021 was used to verify its predictive ability.

**Result:**

A total of 24 articles were included in the meta-analysis. The model includes six risk factors: age, hemorrhagic manifestations, encephalopathy, Scr and BUN. The analysis of lasso regression and multivariate logistic regression shows that model score is an independent risk factor (OR = 1.032, 95% CI 1.002–1.063, *p* = 0.034). The model had an area under the curve (AUC) of 0.779 (95% CI 0.669–0.889, *P*<0.001). The validation cohort was divided into four risk groups with cut-off values. Compared with the low-medium risk group, the mortality rate of high-risk and very high-risk patients was more significant (RR =5.677, 95% CI 4.961–6.496, *P*<0.001).

**Conclusion:**

The prediction model for the fatal outcome of SFTS patients has shown positive outcomes.

**Systematic review registration:**https://www.crd.york.ac.uk/prospero/ (CRD42023453157).

## Introduction

Severe fever with thrombocytopenia syndrome (SFTS) is an acute infectious disease caused by the Severe fever with thrombocytopenia syndrome virus (SFTSV), which is mainly transmitted through tick bites and direct contact with the bodily fluids of SFTS patients (Li et al., 2018). Raising domestic animals is also one of the most important ways to transmit SFTSV in China (Sun et al., 2015). According to reports, there were also reported cases of cat-to-human transmission in Japan (Kida et al., 2019; Yamanaka et al., 2020). The disease was first reported by Chinese scholars in 2010 (Yu et al., 2011). By the end of 2019, confirmed cases have been reported in 25 provinces in China (Li J. et al., 2021). Furthermore, there were also reports of SFTS diagnoses in Japan, South Korea and Vietnam (Yu et al., 2011; Tran et al., 2019; Miao et al., 2021). The typical features of SFTS include high fever, thrombocytopenia, systemic infection symptoms, and the potential development of multiple organ dysfunction syndrome (MODS) in some patients (Miao et al., 2021). The disease progresses rapidly in SFTS patients, with a high mortality rate of 12%–30% (Miao et al., 2021; Li et al., 2021a). Due to its high lethality and potential for endemic or outbreak with expanding affected areas, Severe Fever with Thrombocytopenia Syndrome (SFTS) was classified as a nationally reportable disease in China in 2010 (General Office of the Ministry of Health of the People’s Republic of China, 2010) and was listed by the World Health Organization as one of the top 10 priority infectious diseases in the 2018 annual review of the Blueprint list (World Health Organization, 2017). Although some studies have confirmed the efficacy and safety of favipiravir in the treatment of SFTS (Suemori et al., 2021; Yuan et al., 2021; Li et al., 2021a), specific antiviral treatments and effective vaccines are still lacking (Miao et al., 2021). Therefore, early assessment of the patients’ prognosis is crucial for disease management and improving clinical outcomes.

Numerous studies have investigated the risk factors for fatal outcomes in SFTS patients, encompassing clinical symptoms, signs, and laboratory tests. However, the limited number of cases included in each study and the diverse study cohorts from various regions contribute to the poor consistency among these studies. This systematic review and meta-analysis aim to comprehensively evaluate the identified risk factors for mortality in SFTS reported in previous studies. The study also aims to establish a fatal outcome prediction model that can assist clinicians in the early detection of high-risk groups and facilitate personalized interventions, ultimately improving the prognosis of patients.

## Methods

The protocol of this systematic review was registered with PROSPERO (CRD42023453157).

### Literature screening

We conducted a comprehensive search in four databases, including PubMed, Web of Science, Embase, and the Cochrane Library, to identify relevant literature up to April 4, 2023. The search terms used in this study included “SFTS,” “severe fever with thrombocytopenia syndrome,” “bunyavirus,” and “outcome.” Two authors independently screened the search results and evaluated the quality of the literature using the Newcastle-Ottawa scale. The detailed search strategy and screening criteria are available in the Supplementary material.

### Data extraction

Data were independently extracted by two authors from the included literature. The following information was extracted from each article: first author, publication year, region, study type, sample size, patient age, the number of survivors and deceased, risk factors and corresponding risk ratios (RRs), and 95% confidence intervals. The definitions of symptoms, signs, and abbreviations are detailed in the Supplementary material.

### Validation cohort

From January 2021 to December 2021, 206 confirmed SFTS patients who visited Yantai Qishan Hospital were selected as the verification cohort. The exclusion criteria included other pathogenic infections, blood system diseases, autoimmune diseases, no history of radiotherapy or chemotherapy, and no blood product transfusion within the past two weeks. The patient’s case information and clinical data were complete. A total of 194 patients were ultimately included in the verification cohort, and they were further divided into a survival group and a deceased group based on clinical outcomes.

### Meta-analysis

We extracted the relative risk and 95% CI of each independent risk factor for mortality in SFTS patients and calculated pooled RRs using either a random-effects model or a fixed-effects model. The studies were weighted according to the inverse of the variance of the RRs based on an estimate of statistical size. Heterogeneity was assessed by the Cochrane Q test and measured by the I^2^ value. If I^2^ exceeded 50% or *p* < 0.10, it indicated significant heterogeneity among studies and a random-effects model was used; otherwise, a fixed-effects model was used (Tufanaru et al., 2015). Subgroup analysis was conducted based on variable type or changes in continuous variables. Sensitivity analysis was conducted by sequentially omitting individual studies to test the robustness of the results (Devillé et al., 2002). All analyses were performed using Stata software, version 16.0.

### Model development

First, statistically significant risk factors were identified through the meta-analysis. The RR and 95% CI for each risk factor were extracted and combined. To enhance clinical relevance, the pooled RRs of certain risk factors were selected from subgroup analysis or sensitivity analysis. Then, the corresponding coefficients β were calculated based on pooled RRs, multiplied by 10 and rounded to one decimal place for score calculation. Finally, all risk factors in the predictive model were stratified, and the corresponding scores were matched for each level. The sum of the scores in the model was the total score (Sullivan et al., 2004).

### Model validation

The model scores were applied to the validation cohort and analyzed using lasso regression, along with other baseline data (Sauerbrei et al., 2007). Multivariate logistic regression analysis was conducted on the non-zero coefficient variable at log(λ) = lambda min while adjusting for confounding factors. Variables with *p* < 0.05 were independent risk factors for mortality in SFTS patients. The receiver operating characteristic (ROC) curve was plotted and the AUC was calculated to evaluate the specificity and sensitivity of the prediction model. A higher AUC value closer to 1 indicates superior performance of the model (Cook, 2007). The Hosmer-Lemeshow test was used to assess the goodness of fit of the prediction model, and a calibration curve was plotted. *p* > 0.05 indicated no statistical difference between the predicted and actual values in the model. Additionally, we assessed the benefit at various threshold probabilities in the validation cohort and conducted a decision curve analysis to evaluate the clinical usefulness of the risk prediction model (Vickers et al., 2008). Furthermore, we used x-tile software to determine the optimal cut-off value and divided patients into different risk groups based on this value. Kaplan–Meier curves were plotted to compare survival differences among groups.

The statistical analyses were performed using SPSS 25.0, R Studio 3.3.3, x-tile 3.6.1, and GraphPad Prism 9.3.0 software.

## Result

### Literature screening

**Figure 1:**
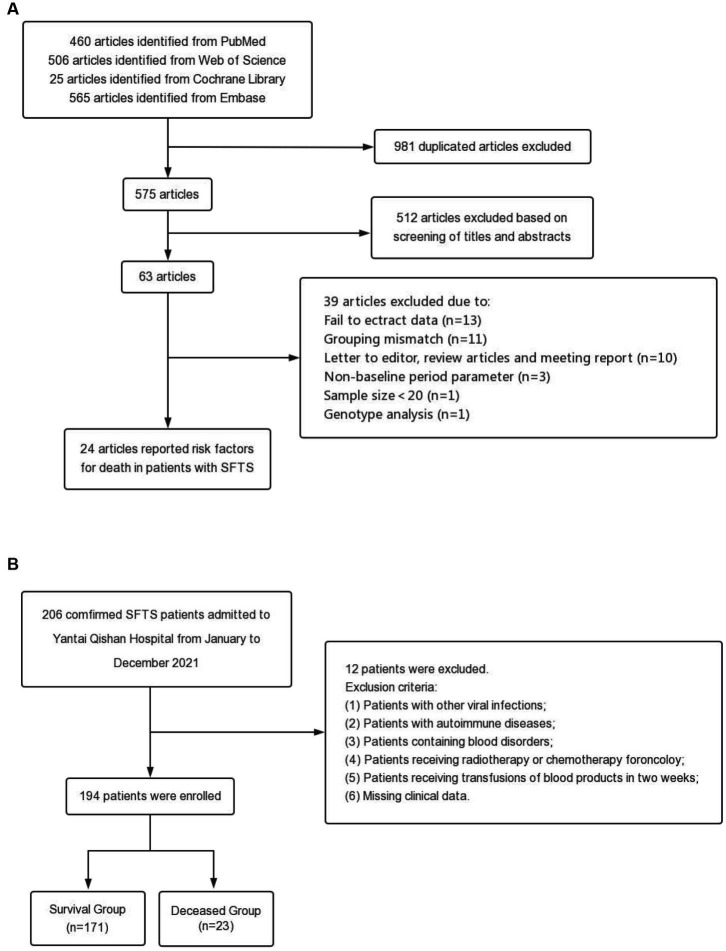
Flow chart of this study. **(A)** Flow diagram outlining the literature search and study selection for risk factors of death in patients with SFTS. **(B)** Process for the selection of patients in the validation cohort.

The process and results of the literature search and screening are shown in Figure 1A. A total of 1,556 articles were retrieved from the four databases, including 981 duplicate articles. After reading titles and abstracts, 512 irrelevant articles were excluded. After reading the full text according to the selection criteria, 39 articles were excluded. Finally, 24 articles were included in the further meta-analysis. The relevant information for all articles is shown in Supplementary Table S1. According to the Newcastle-Ottawa scale, the final scores of the included studies were all above 5 points, and the scores are detailed in Supplementary Table S2.

The 24 studies included a total of 4,793 SFTS patients, of whom 930 died. 49 risk factors were obtained from the included cohort studies, including age, hemorrhagic manifestations, neurological symptoms and signs, WBC, AST, APTT, etc. The detailed information on all risk factors is provided in Supplementary Table S3.

### Validation cohort

The process and results of patient selection for the validation cohort are shown in Figure 1B. A total of 194 SFTS patients were included in the validation cohort, of whom 47.94% were female, with a mean age of 62.39 ± 11.85 years and a median hospital stay of 10.0 days (IQR 6.0–13.0 days). Patients were divided into two groups based on clinical outcomes, with 171 patients in the survival group and 23 patients in the deceased group. The incidence of hemorrhagic manifestations and neurological symptoms in the deceased group was higher than in the survival group (hemorrhagic manifestations: 1.2% in the survival group and 17.4% in the deceased group; neurological symptoms: 8.8% in the survival group and 34.8% in the deceased group). There were significant differences in the laboratory indexes of LYM%, PLT, MPV, LDH, CK, CK-MB, ALT, AST, TBIL, DBIL, ALB, GGT, ALP, BUN, Scr, Ca, PCT, and CRP among the two groups. The baseline characteristics of patients in the validation cohorts are shown in Supplementary Table S4.

### Model development

**Figure 2:**
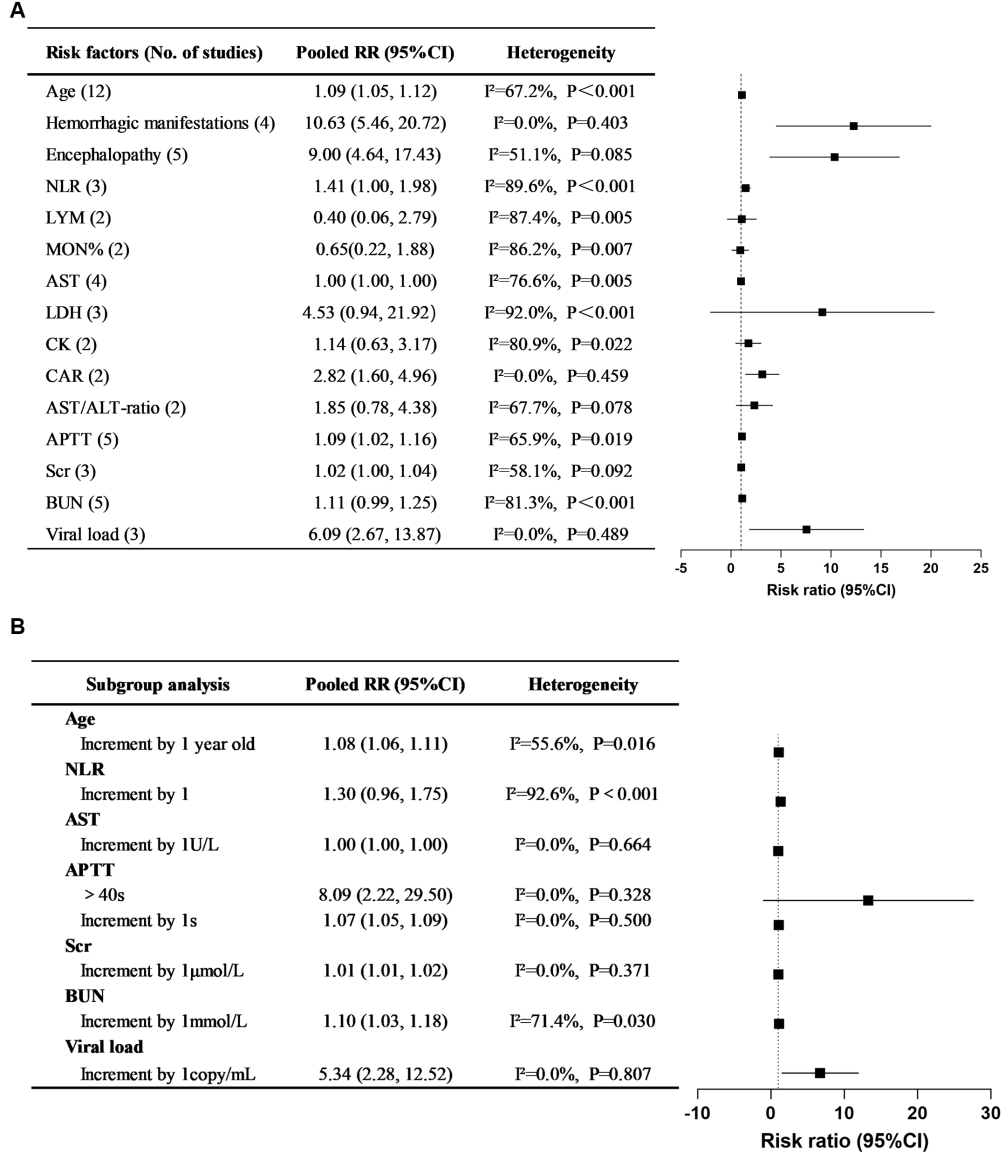
Pooled RRs of risk factors for mortality in SFTS patients and their corresponding 95% CIs in systematic reviews and meta-analyses. **(A)** Overall pooled RRs and their 95% CIs of SFTS mortality risk factors. **(B)** Subgroup study for the risk factors of SFTS mortality.

A total of 49 risk factors were obtained from the 24 cohort studies, and 15 of them were eligible for further meta-analysis. The forest plot of each factor is shown in Figure 2, and the forest plots of each risk factor and corresponding subgroup or sensitivity analysis can be found in Supplementary Figures S1–S15. Details of the risk factors that were statistically significant in the meta-analysis are presented in Supplementary Table S5. Based on the data analysis results and clinical practice, NLR, CAR, and viral load were not included in this risk prediction model. Variables with meaningless pooled RR were excluded, and the final risk factors included in the model were age (RR 1.08, 95% CI 1.06–1.11), hemorrhagic manifestations (RR 10.63, 95% CI 5.46–20.72), encephalopathy (RR 9.00, 95% CI 4.64–17.43), APTT (RR 1.07, 95% CI 1.05–1.09), Scr (RR 1.01, 95% CI 1.01–1.02), and BUN (RR 1.10, 95% CI 1.03–1.18). Based on clinical practice, we have developed a mortality risk prediction model for SFTS patients, as shown in Table 1. This prediction model is recommended for early assessment of the prognosis in SFTS patients aged 18 and above, primarily targeting the Asian population.

**Table 1:** SFTS mortality risk prediction model.

Risk factor	Category	Point
Age (years)	<30	0
	30 ~ 39	8
	40 ~ 49	16
	50 ~ 59	24
	60 ~ 69	32
	≥70	40
Hemorrhagic manifestations	No	0
	Yes	24
Encephalopathy	No	0
	Yes	22
APTT (s)	≤37	0
	38 ~ 47	7
	48 ~ 57	14
	58 ~ 67	21
	>67	28
Scr (μmol/L)	≤133	0
	134 ~ 233	10
	234 ~ 333	20
	>333	30
BUN (mmol/L)	≤7.1	0
	7.1 ~ 17.1	10
	>17.1	20

APTT, activated partial thromboplastin time; BUN, blood urea nitrogen; Scr, serum creatinine.

### Model validation

We calculated the scores of each patient in the validation cohort based on the model and conducted Lasso regression analysis (Supplementary Figure S16) along with other baseline data. We found that the model score, CK, CKMB, AST, DBIL, ALB, ALP, APTT, and CRP were important factors for predicting the mortality risk in SFTS patients. Multivariate logistic regression analysis of these 9 factors showed that the model score (OR = 1.032, 95% CI 1.002–1.063, *p* = 0.034), CKMB (OR = 1.010, 95% CI 1.001–1.020, *p* = 0.037), AST (OR = 1.003, 95% CI 1.001–1.005, *p* < 0.001), and CRP (OR = 1.052, 95% CI 1.004–1.102, *p* = 0.035) were independent risk factors for mortality in SFTS patients (Table 2). Supplementary Figure S7 shows that the OR of the model score is still statistically significant after adjusting for gender, symptoms, signs, and medical history. It indicates that the model can stably predict the mortality risk in SFTS patients.

**Table 2:** Multivariate logistic regression analysis of prognostic risk factors in patients with SFTS.

Risk factors	OR	95% CI	*p* value
Model Scores	1.032	1.002–1.063	0.034
CKMB	1.010	1.001–1.020	0.037
AST	1.003	1.001–1.005	<0.001
CRP	1.052	1.004–1.102	0.035

CKMB, creatine kinase isoenzyme; AST, aspertate aminotransferase; CRP, C-reactive protein.

**Figure 3:**
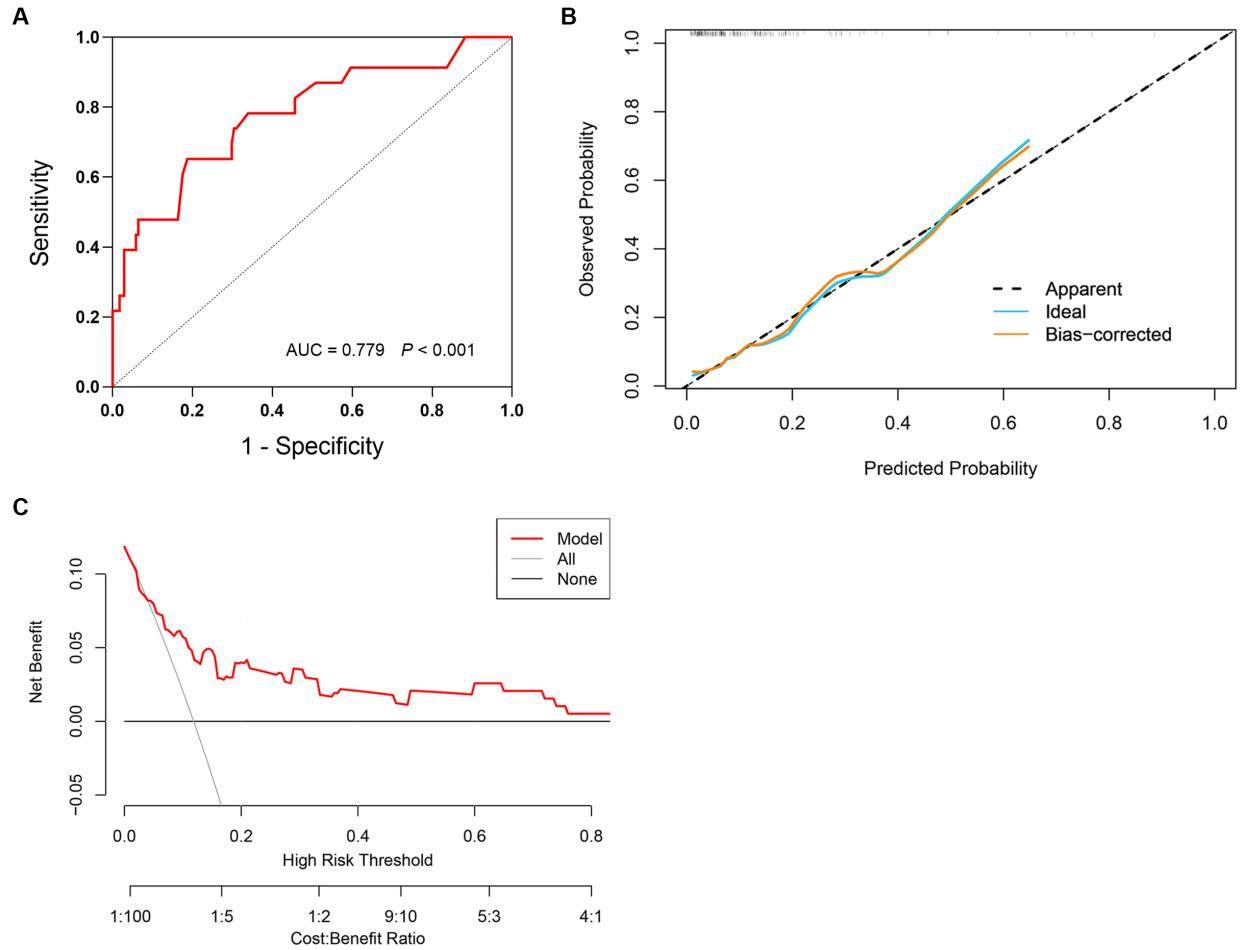
Validation of prediction model for mortality risk in SFTS patients. **(A)** The prediction model has an AUC of 0.779 (95% CI 0.669–0.889, *p* < 0.001). **(B)** Calibration curves for the prediction model of mortality risk. **(C)** Decision curve analysis for the prediction model of mortality risk.

According to the ROC curve (Figure 3A), the model had an AUC of 0.779 (95% CI 0.669–0.889, *p* < 0.001), indicating good sensitivity and specificity. The Hosmer-Lemeshow test showed *p* = 0.269, indicating high consistency between the predicted outcomes of the model and the actual outcomes of the cohort patients, as well as a good fit of the model. The same conclusion can also be drawn from the calibration curve (Figure 3B). Additionally, Figure 3C shows the clinical decision curve of the prediction model, indicating that using this model to predict the mortality risk in SFTS patients before clinical intervention has more benefits than treating all patients or not treating them at all.

**Figure 4:**
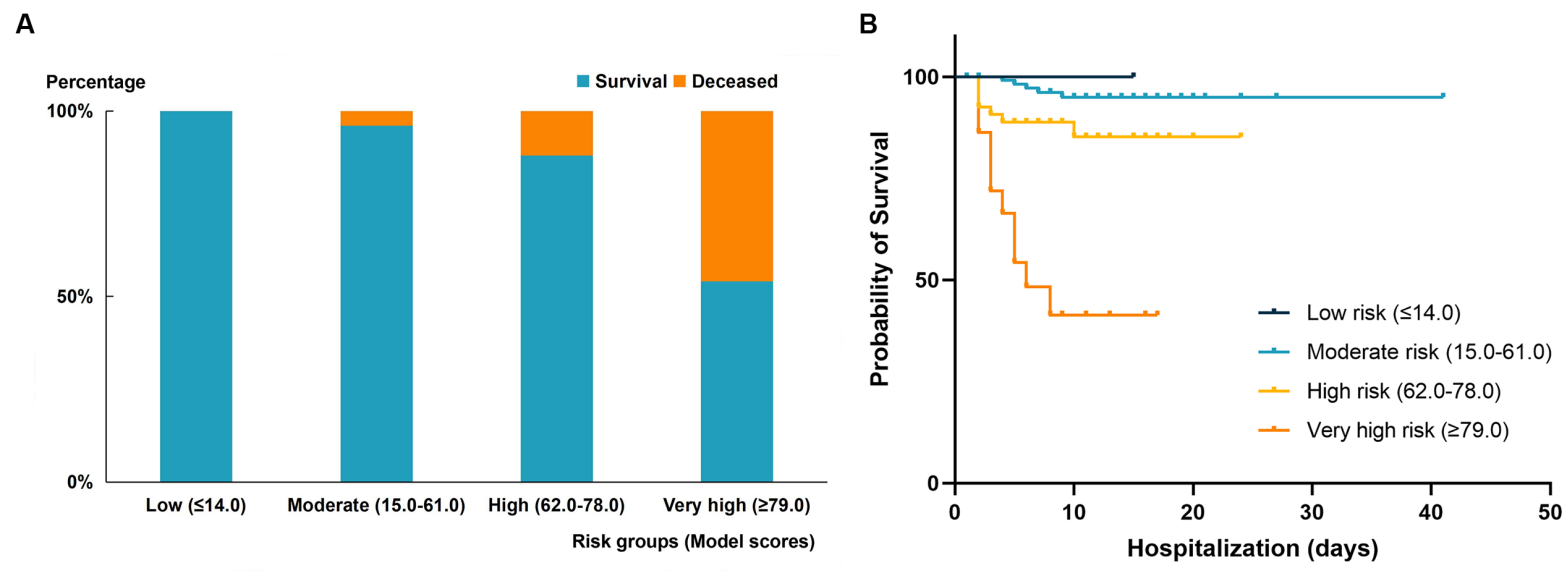
Comparison of outcomes in different mortality risk groups of the validation cohort. **(A)** Mortality rate in the four risk groups stratified by risk score in the validated cohort. **(B)** Kaplan–Meier curve of survival endpoint for each risk group. High-risk group: RR 2.775 (95% CI 0.921–8.359, *P* < 0.001); very high-risk group: 10.358 (95% CI 3.962–27.079, *P* < 0.001).

Using the x-tile software, the score cut-off value obtained can divide the validation cohort patients into four risk level groups: low risk (≤14), moderate risk (15–61), high risk (62–78), and very high risk (≥79). The mortality rates of the four groups of patients were 0, 4.0, 12.0, and 46.0%, respectively. The comparison of survival and mortality rates in each group can be seen in Figure 4A. Additionally, Figure 4B shows the Kaplan–Meier curves for each group. Compared to the moderate and low-risk groups, the patients in the very high-risk group had RR of 10.358 (95% CI 3.962–27.079, *p* < 0.001), and the mortality risk of patients in the high-risk and very high-risk groups was more significant (RR = 5.677, 95% CI 4.961–6.496, *p* < 0.001).

## Discussion

### Main findings

Through meta-analysis, 15 independent risk factors for mortality in SFTS patients were identified from 24 related studies, including age, hemorrhagic manifestations, encephalopathy, LYM, MON%, AST, AST/ALT ratio, LDH, CK, APTT, Scr, BUN, CAR, NLR, and viral load. However, further analysis revealed that the indicators MON%, AST, AST/ALT ratio, LDH, and CK did not show statistical significance in Pooled RRs and were subsequently excluded. Based on the statistical results and clinical conditions, a fatal outcome prediction model for SFTS patients was established by incorporating 6 risk factors, including age, hemorrhagic manifestations, encephalopathy, APTT, BUN, and Scr. The model score, identified through multivariate logistic regression, is an independent risk factor for mortality in SFTS patients. Early utilization of this model to assess the risk of mortality in SFTS patients can facilitate personalized interventions, thereby effectively improving their prognosis.

The study’s findings indicated a positive correlation between increasing age and the risk of death in SFTS patients. Li et al. (2018) emphasized that advanced age was an independent risk factor for fatal outcomes in SFTS patients. This may be related to the lower immune function, higher infection rate, and higher incidence of complications in elderly people (Xu et al., 2018). Metcalf et al. (2017) suggested that transcriptional changes occur in monocyte subpopulations during the aging process, leading to upregulation of pattern recognition receptor signaling and excessive cytokine production, making monocytes in elderly individuals more susceptible to SFTSV infection.

Hemorrhagic manifestations were defined as skin ecchymosis, oral gingival bleeding, gastrointestinal bleeding and pulmonary bleeding (He et al., 2021). And encephalopathy was defined as an altered mental status that persisted for more than 24 h, including lethargy, irritability, or a change in personality and behavior (Wang et al., 2019). The study revealed that hemorrhagic manifestations and encephalopathy were significant indicators for predicting clinical outcomes in patients with SFTS. The risk of mortality in patients with hemorrhagic manifestations or encephalopathy was 9.63 and 8 times higher than that of patients without these symptoms, respectively. Moreover, prolonged APTT is also an important factor in predicting fatal outcomes in SFTS patients. When APTT increases by 1 s, the risk of mortality in SFTS patients increases by 7%. Wang et al. (2021) suggested that hemorrhagic manifestations and prolonged APTT are independent risk factors for mortality in SFTS patients. However, the exact reason behind the prolongation of APTT remains unclear. Some scholars have proposed that acute liver injury caused by SFTSV leads to a decrease in the synthesis of coagulation factors, resulting in coagulation disorders that can ultimately cause DIC and the fatal outcome of patients (Zhang et al., 2012). On the one hand, the decrease in platelets and coagulation factors in SFTS patients can lead to a tendency for systemic hemorrhage. On the other hand, SFTSV infection can trigger enhanced complement activation, which is related to the occurrence and progression of DIC in patients (Li et al., 2021b), ultimately leading to death. In addition, the mechanism of neurological symptoms in SFTS patients is still unclear, but it is believed to be related to direct invasion of the central nervous system by SFTSV as well as increased levels of IL-8 and MCP-1 in the cerebrospinal fluid (Park et al., 2018).

In our study, renal function was also closely related to the clinical outcomes of SFTS patients. When BUN increased by 1 mmol/L, the risk of mortality in patients increased by 10%, while when Scr increased by 1 umol/L, the risk of mortality increased by 1%. Previous studies have reported that the kidney may be one of the targets of SFTSV attack, and severe renal function damage can lead to mortality in SFTS patients (Jin et al., 2012). BUN and Scr are commonly used indicators for assessing renal function in clinical practice, so monitoring changes in these indicators early can help evaluate the patient’s prognosis.

In our meta-analysis, the increase in viral load, NLR and CAR will increase the risk of mortality in patients by 509%, 41%, and 182%, respectively. However, we did not include these three risk factors in the model. The first reason is that there is significant heterogeneity among NLR-related studies, and this heterogeneity could not be eliminated or reduced through subgroup analysis or sensitivity analysis. Secondly, not all medical institutions have SFTS viral load detection technology, and different institutions use different units to measure viral load detection results. Thirdly, NLR and CAR are complex composite indicators with few practical clinical applications and complicated calculations. Finally, we established a mortality risk prediction model that included CAR. We defined a score of 10 points as being assigned when CAR>1. Compared with the model without CAR, the inclusion of CAR did not significantly improve the predictive ability of the model.

### Implications, strengths and limitations

The scoring model was externally validated in a cohort of 194 SFTS patients. Through lasso regression and multivariate logistic regression analysis, we confirmed that the model score is an independent risk factor of mortality for SFTS patients. Additionally, the ROC curve demonstrated that the model had good prediction ability of mortality risk in the validation cohort (AUC = 0.779, 95% CI 0.669–0.889, *P*<0.001). The calibration curve and clinical decision curve further confirmed the model’s strong fit and clinical applicability. Finally, based on the analysis results, we established risk level stratification using cut-off values of 14, 61, and 78, dividing SFTS patients into relatively low, medium, high, and very high risk groups. Compared with patients in the medium-low risk group, patients in the very high risk group had a significantly higher risk of death, about 9.36 times higher.

In summary, this mortality risk prediction model for patients with SFTS is based on numerous previous research results and has been validated for sensitivity, specificity and clinical applicability in a certain sample size of the validation cohort. It can effectively assist clinicians in identifying high risk patients early on and taking reasonable treatment measures timely to maximize improvement in patient prognosis. Furthermore, the risk prediction model we designed is user friendly as it does not require complex calculations, and the laboratory parameters included in the model do not necessitate high detection equipment. Therefore, it is convenient to apply it in clinical practice.

However, our study has the following limitations that should be acknowledged. Firstly, although previous studies have shown that liver function damage is an independent risk factor for mortality in SFTS patients (Yu et al., 2011), but laboratory indicators that reflect patient liver function were not included in the model due to the lack of statistical significance in pooled RRs. This may be due to the large heterogeneity among studies caused by the limited sample size and different sources of samples. Secondly, the validation cohort we used had a limited sample size and was obtained from a single source, while the prevalence of SFTS has significant regional differences (Zhang et al., 2021), which may introduce regional bias and affect the generalizability of our results. Lastly, baseline mortality risk prediction may vary depending on the study population. Hence, future studies could consider calibrating the model based on population type to enhance the accuracy of the death risk prediction model.

## Conclusion

Based on a systematic review and meta-analysis, we developed an early SFTS patient mortality risk prediction model. The model incorporates clinical manifestations and laboratory indicators of SFTS patients, including age, hemorrhagic manifestations, encephalopathy, APTT, BUN and Scr. Additionally, the model’s effectiveness in assessing the risk of mortality in SFTS patients has been validated by an external cohort.

## Data availability statement

The original contributions presented in the study are included in the article/Supplementary material, further inquiries can be directed to the corresponding author.

## Author contributions

ZL: Conceptualization, Data curation, Writing – original draft, Writing – review & editing. ZJ: Data curation, Formal analysis, Writing – original draft, Writing – review & editing. LZ: Formal analysis, Methodology, Writing – original draft, Writing – review & editing. XX: Resources, Writing – review & editing. CZ: Resources, Writing – review & editing. YX: Methodology, Writing – review & editing. WZ: Resources, Writing – review & editing. LL: Project administration, Resources, Writing – review & editing. ZC: Conceptualization, Funding acquisition, Project administration, Supervision, Writing – review & editing.

## Supplementary material

SUPPLEMENTARY FIGURE S1 Age. **(A)** Association of age with mortality of SFTS patients. **(B)** Subgroup (increment by 1 year) analysis of association of age with mortality of SFTS patients.

SUPPLEMENTARY FIGURE S2 Hemorrhagic manifestations. Association of hemorrhagic manifestations with mortality of SFTS patients.

SUPPLEMENTARY FIGURE S3 Encephalopathy. Association of encephalopathy with mortality of SFTS patients.

SUPPLEMENTARY FIGURE S4 The ratio of neutrophil to lymphocyte. **(A)** Association of NLR with mortality of SFTS patients; **(B)** Subgroup (increment by 1) analysis of association of NLR with mortality of SFTS patients. NLR: the ratio of neutrophil to lymphocyte.

SUPPLEMENTARY FIGURE S5 Association of lymphocyte with mortality of SFTS patients.

SUPPLEMENTARY FIGURE S6 Association of percentage of monocytes with mortality of SFTS patients.

SUPPLEMENTARY FIGURE S7 Aspertate aminotransferase. **(A)** Association of aspertate aminotransferase with mortality of SFTS patients; **(B)** Subgroup (increment by 1U/L) analysis of association of aspertate aminotransferase with mortality of SFTS patients.

SUPPLEMENTARY FIGURE S8 Lactate dehydrogenase. Association of lactate dehydrogenase with mortality of SFTS patients.

SUPPLEMENTARY FIGURE S9 AST/ALT-ratio. Association of AST/ALT-ratio with mortality of SFTS patients.

SUPPLEMENTARY FIGURE S10 Creatine kinase. Association of creatine kinase with mortality of SFTS patients.

SUPPLEMENTARY FIGURE S11 The ratio of C-reactive protein to albumin. Association of CAR with mortality of SFTS patients. CAR: the ratio of C-reactive protein to albumin.

SUPPLEMENTARY FIGURE S12 Activated partial thromboplastin time. **(A)** Association of APTT with mortality of SFTS patients; **(B)** Subgroup analysis of association of APTT with mortality of SFTS patients. APTT: Activated partial thromboplastin time.

SUPPLEMENTARY FIGURE S13 Serum creatinine. **(A)** Association of serum creatinine with mortality of SFTS patients; **(B)** Subgroup (increment by 1 μmol/L) analysis of association of serum creatinine with mortality of SFTS patients.

SUPPLEMENTARY FIGURE S14 Blood urea nitrogen. **(A)** Association of blood urea nitrogen with mortality of SFTS patients; **(B)** Subgroup (increment by 1 mmol/L) analysis of association of blood urea nitrogen with mortality of SFTS patients.

SUPPLEMENTARY FIGURE S15 Viral load. **(A)** Association of viral load with mortality of SFTS patients; **(B)** Subgroup (increment by 1 copy/mL) analysis of association of viral load with mortality of SFTS patients.

SUPPLEMENTARY FIGURE S16 Determination of prognostic risk factors in SFTS patients by the LASSO analysis. **(A)** LASSO coefficient profiles of the 35 risk factors; **(B)** Tuning parameter (λ) selection in the LASSO model used 10-fold cross-validation via minimum criteria.

## References

[ref1] Cook N. R. (2007). Use and misuse of the receiver operating characteristic curve in risk prediction. Circulation 115, 928–935. doi: 10.1161/CIRCULATIONAHA.106.672402 17309939

[ref2] Devillé W. L. Buntinx F. Bouter L. M. Montori V. M. de Vet H. C. van der Windt D. A. . (2002). Conducting systematic reviews of diagnostic studies: didactic guidelines. BMC Med. Res. Methodol. 2:9. doi: 10.1186/1471-2288-2-9 12097142 PMC117243

[ref3] General Office of the Ministry of Health of the People’s Republic of China (2010) Notice on Issuing Guidelines for the Prevention and Treatment of Fever with Thrombocytopenia Syndrome (2010 Edition). Available at: https://www.gov.cn/gzdt/2010-10/09/content_1718261.htm (Accessed August 9, 2023).

[ref4] He F. Zheng X. Zhang Z. (2021). Clinical features of severe fever with thrombocytopenia syndrome and analysis of risk factors for mortality. BMC Infect. Dis. 21:1253. doi: 10.1186/s12879-021-06946-3, PMID: 34906106 PMC8669668

[ref5] Jin C. Liang M. Ning J. Gu W. Jiang H. Wu W. . (2012). Pathogenesis of emerging severe fever with thrombocytopenia syndrome virus in C57/BL6 mouse model. Proc. Natl. Acad. Sci. U. S. A. 109, 10053–10058. doi: 10.1073/pnas.1120246109, PMID: 22665769 PMC3382536

[ref6] Kida K. Matsuoka Y. Shimoda T. Matsuoka H. Yamada H. Saito T. . (2019). A case of cat-to-human transmission of severe fever with thrombocytopenia syndrome virus. Jpn. J. Infect. Dis. 72, 356–358. doi: 10.7883/yoken.JJID.2018.526, PMID: 31366857

[ref7] Li H. Jiang X. M. Cui N. Yuan C. Zhang S. F. Lu Q. B. . (2021a). Clinical effect and antiviral mechanism of T-705 in treating severe fever with thrombocytopenia syndrome. Signal Transduct. Target. Ther. 6:145. doi: 10.1038/s41392-021-00541-3, PMID: 33859168 PMC8050330

[ref8] Li H. Li X. Lv S. Peng X. Cui N. Yang T. . (2021b). Single-cell landscape of peripheral immune responses to fatal SFTS. Cell Rep. 37:110039. doi: 10.1016/j.celrep.2021.110039 34818556

[ref9] Li J. Li S. Yang L. Cao P. Lu J. (2021). Severe fever with thrombocytopenia syndrome virus: a highly lethal bunyavirus. Crit. Rev. Microbiol. 47, 112–125. doi: 10.1080/1040841X.2020.1847037, PMID: 33245676

[ref10] Li H. Lu Q. B. Xing B. Zhang S. F. Liu K. Du J. . (2018). Epidemiological and clinical features of laboratory-diagnosed severe fever with thrombocytopenia syndrome in China, 2011-17: a prospective observational study. Lancet Infect. Dis. 18, 1127–1137. doi: 10.1016/S1473-3099(18)30293-7, PMID: 30054190

[ref11] Metcalf T. U. Wilkinson P. A. Cameron M. J. Ghneim K. Chiang C. Wertheimer A. M. . (2017). Human monocyte subsets are transcriptionally and functionally altered in aging in response to pattern recognition receptor agonists. J. Immunol. 199, 1405–1417. doi: 10.4049/jimmunol.1700148, PMID: 28696254 PMC5548610

[ref12] Miao D. Liu M. J. Wang Y. X. Ren X. Lu Q. B. Zhao G. P. . (2021). Epidemiology and ecology of severe fever with thrombocytopenia syndrome in China, 2010–2018. Clin. Infect. Dis. 73, e3851–e3858. doi: 10.1093/cid/ciaa1561, PMID: 33068430 PMC8664468

[ref13] Park S. Y. Kwon J. S. Kim J. Y. Kim S. M. Jang Y. R. Kim M. C. . (2018). Severe fever with thrombocytopenia syndrome-associated encephalopathy/encephalitis. Clin. Microbiol. Infect. 24, 432.e1–432.e4. doi: 10.1016/j.cmi.2017.09.002, PMID: 28899841

[ref14] Sauerbrei W. Royston P. Binder H. (2007). Selection of important variables and determination of functional form for continuous predictors in multivariable model building. Stat. Med. 26, 5512–5528. doi: 10.1002/sim.3148, PMID: 18058845

[ref15] Suemori K. Saijo M. Yamanaka A. Himeji D. Kawamura M. Haku T. . (2021). A multicenter non-randomized, uncontrolled single arm trial for evaluation of the efficacy and the safety of the treatment with favipiravir for patients with severe fever with thrombocytopenia syndrome. PLoS Negl. Trop. Dis. 15:e0009103. doi: 10.1371/journal.pntd.0009103, PMID: 33617533 PMC7899362

[ref16] Sullivan L. M. Massaro J. M. D’Agostino R. B. Sr. (2004). Presentation of multivariate data for clinical use: the Framingham study risk score functions. Stat. Med. 23, 1631–1660. doi: 10.1002/sim.1742 15122742

[ref17] Sun J. M. Zhang Y. J. Gong Z. Y. Zhang L. Lv H. K. Lin J. F. . (2015). Seroprevalence of severe fever with thrombocytopenia syndrome virus in southeastern China and analysis of risk factors. Epidemiol. Infect. 143, 851–856. doi: 10.1017/S0950268814001319, PMID: 24866248 PMC4411641

[ref18] Tran X. C. Yun Y. Van An L. Kim S. H. Thao N. T. P. Man P. K. C. . (2019). Endemic severe fever with thrombocytopenia syndrome, Vietnam. Emerg. Infect. Dis. 25, 1029–1031. doi: 10.3201/eid2505.181463, PMID: 31002059 PMC6478219

[ref19] Tufanaru C. Munn Z. Stephenson M. Aromataris E. (2015). Fixed or random effects meta-analysis? Common methodological issues in systematic reviews of effectiveness. Int. J. Evid. Based Healthc. 13, 196–207. doi: 10.1097/XEB.0000000000000065 26355603

[ref20] Vickers A. J. Cronin A. M. Elkin E. B. Gonen M. (2008). Extensions to decision curve analysis, a novel method for evaluating diagnostic tests, prediction models and molecular markers. BMC Med. Inform. Decis. Mak. 8:53. doi: 10.1186/1472-6947-8-53 19036144 PMC2611975

[ref21] Wang L. Wan G. Shen Y. Zhao Z. Lin L. Zhang W. . (2019). A nomogram to predict mortality in patients with severe fever with thrombocytopenia syndrome at the early stage-a multicenter study in China. PLoS Negl. Trop. Dis. 13:e0007829. doi: 10.1371/journal.pntd.0007829, PMID: 31765414 PMC6934327

[ref22] Wang L. Wan G. Shen Y. Zhao Z. Lin L. Zhang W. . (2021). Clinical manifestations of death with severe fever and thrombocytopenia syndrome: a meta-analysis and systematic review. J. Med. Virol. 93, 3960–3968. doi: 10.1002/jmv.26518, PMID: 32930400

[ref23] World Health Organization. (2017) Annual review of diseases prioritized under the Research and Development Blueprint; 2017. Available at: https://cdn.who.int/media/docs/default-source/blue-print/first-annual-review-of-diseases-prioritized-under-r-and-d-blueprint.pdf?sfvrsn=1f6b5da0_4 (Accessed August 9, 2023)

[ref24] Xu X. Sun Z. Liu J. Zhang J. Liu T. Mu X. . (2018). Analysis of clinical features and early warning indicators of death from severe fever with thrombocytopenia syndrome. Int. J. Infect. Dis. 73, 43–48. doi: 10.1016/j.ijid.2018.05.013, PMID: 29859247

[ref25] Yamanaka A. Kirino Y. Fujimoto S. Ueda N. Himeji D. Miura M. . (2020). Direct transmission of severe fever with thrombocytopenia syndrome virus from domestic cat to veterinary personnel. Emerg. Infect. Dis. 26, 2994–2998. doi: 10.3201/eid2612.191513, PMID: 33219655 PMC7706950

[ref26] Yu X. J. Liang M. F. Zhang S. Y. Liu Y. Li J. D. Sun Y. L. . (2011). Fever with thrombocytopenia associated with a novel bunyavirus in China. N. Engl. J. Med. 364, 1523–1532. doi: 10.1056/NEJMoa1010095, PMID: 21410387 PMC3113718

[ref27] Yuan Y. Lu Q. B. Yao W. S. Zhao J. Zhang X. A. Cui N. . (2021). Clinical efficacy and safety evaluation of favipiravir in treating patients with severe fever with thrombocytopenia syndrome. EBioMedicine 72:103591. doi: 10.1016/j.ebiom.2021.103591 34563924 PMC8479638

[ref28] Zhang Y. Z. He Y. W. Dai Y. A. Xiong Y. Zheng H. Zhou D. J. . (2012). Hemorrhagic fever caused by a novel Bunyavirus in China: pathogenesis and correlates of fatal outcome. Clin. Infect. Dis. 54, 527–533. doi: 10.1093/cid/cir804, PMID: 22144540

[ref29] Zhang Y. Miao W. Xu Y. Huang Y. (2021). Severe fever with thrombocytopenia syndrome in Hefei: clinical features, risk factors, and ribavirin therapeutic efficacy. J. Med. Virol. 93, 3516–3523. doi: 10.1002/jmv.26544, PMID: 32965706

