# Supplementary material for: A model based on meta-analysis to evaluate poor prognosis of patients with severe fever with thrombocytopenia syndrome
Source: Front Microbiol. 2024 Jan 8;14:1307960. doi: 10.3389/fmicb.2023.1307960 (PMC10801726; doi:10.3389/fmicb.2023.1307960)
Supplement: Supplementary file 1 [file Data_Sheet_1.docx]

**Supplementary method**

**Search strategy** (up to 2023-04-04)

1. **PubMed**

(SFTS [Title/Abstract] OR “severe fever with thrombocytopenia syndrome” [Title/Abstract] OR “Dabie bandavirus” [Title/Abstract] OR bunyavirus [Title/Abstract]) AND(outcome [Title/Abstract] OR fatal [Title/Abstract] OR non-fatal [Title/Abstract] OR survival [Title/Abstract] OR death [Title/Abstract] OR survivor [Title/Abstract] OR survived [Title/Abstract] OR deceased [Title/Abstract] OR epidemiological [Title/Abstract])

1. **Web of Science**

TS = (SFTS OR "severe fever with thrombocytopenia syndrome" OR "Dabie bandavirus" OR bunyavirus) AND TS = (outcome OR epidemiological OR fatal OR non-fatal OR survival OR death OR survived OR deceased OR prognosis)

1. **Cochrane Library**

((SFTS):ti,ab,kn OR ("severe fever with thrombocytopenia syndrome"):ti,ab,kn OR ("Dabie bandavirus"):ti,ab,kn OR (bunyavirus):ti,ab,kn) AND ((outcome):ti,ab,kn OR (epidemiological):ti,ab,kn OR (survival):ti,ab,kn OR (fatal):ti,ab,kn OR (non-fatal):ti,ab,kn OR (death):ti,ab,kn OR (survived):ti,ab,kn OR (deceased):ti,ab,kn OR (prognosis):ti,ab,kn)

1. **Embase**

((SFTS):ab,ti OR (("severe fever with thrombocytopenia syndrome"):ab,ti) OR (("Dabie bandavirus"):ab,ti) OR ((bunyavirus):ab,ti)) AND ((outcome):ti,ab OR ((epidemiological):ti,ab) OR ((survival):ti,ab) OR ((fatal):ti,ab) OR ((non-fatal):ti,ab) OR ((death):ti,ab) OR ((survived):ti,ab) OR ((deceased):ti,ab) OR ((prognosis):ti,ab))

**Screening criteria**

1. **The inclusion criteria**

*Study*: a. the article had been accepted for publication; b. the study examined the risk factors for mortality in patients with SFTS and reported the risk ratios (RRs) and corresponding 95% confidence limits (CIs) of these risk factors; c. the study provided information and clinical data on SFTS patients, the laboratory parameters analyzed are patients’ baseline data.

*Participants* (meeting one or more of the following criteria): a. the virus was isolated from the patient’s samples; b. SFTSV RNA was detected in the patient’s serum; c. a 4-fold or greater increase in antibody titers was detected between a paired patient serum samples collected from the acute and convalescent phases of infection.

*Outcome*: deceased.

**② The exclusion criteria**

Abstract-only articles, case reports, letters, editorials, systematic reviews, meeting reports, duplicated publications, in vitro studies, studies on animals, study groups are not survival and non-survival, non-baseline period parameters, genotype analyses or treatments, sample size＜20, and articles in which no data were extracted.

**Abbreviated word**

SFTS, severe fever with thrombocytopenia syndrome; MOD, multiple organ dysfunction; SIRS, systemic inflammatory response syndrome; CHD, coronary atherosclerotic heart disease; CRP, C-reactive protein; PCT, procalcitonin; WBC, white blood cell; NEUT, neutrophile granulocyte; LYM, lymphocyte; EOS, eosinophilic granulocyte; BAS, basophilic granulocyte; MON, monocytes; RBC, erythrocyte/red blood cell; HGB, haemoglobin; PLT, thrombocyte; MPV, mean platelet volume; ALB, albumin; ALT, alanine aminotransferase; AST, aspertate aminotransferase; TBIL, total bilirubin; DBIL, direct bilirubin; ALP, alkaline phosphatase; GGT, gamma-glutamyltransferase; GLOB, globulin; LDH, lactate dehydrogenase; CK, creatine kinase; CKMB, creatine kinase isoenzyme; TT, thrombin time; INR, international normalized ratio; APTT, activated partial thromboplastin time; PT, prothrombin time; Glu, glucose; K^+^, kalium; Na^+^, natrium; Ca^2+^, calcium; BUN, blood urea nitrogen; Scr, serum creatinine; UA, uric acid; NLR, the ratio of neutrophil to lymphocyte; CAR, the ratio of C-reactive protein to albumin; PAR, the ratio of platelet to albumin.

**Definitions**

1. Haemorrhagic manifestations included skin ecchymosis, oral gingival bleeding, gastrointestinal bleeding, and pulmonary bleeding.
2. Neurological signs: abnormalities of the cranial nerves, abnormalities of motor system function (such as muscle wasting, muscle tone and power, posture and gait, involuntary movements, and ataxia), abnormalities of sensory function (hypoesthesia and paresthesia), abnormal neural reﬂexes (such as superﬁcial reﬂex, deep reﬂex, pathological reﬂex, and signs of meningeal irritation), and abnormalities of the autonomic system.
3. Disturbance of consciousness: drowsiness, confusion, lethargy, or a severe disturbance of consciousness (no Glasgow Coma Score evaluation was done).
4. APACHE II: Acute Physiology and Chronic Health Evaluation II.
5. Neurologic symptoms: limb tremor/blurred mind/slower reaction, strong decrease consist of drowsiness or frequent muscle convulsion, serious decrease of coma.
6. Respiratory symptoms: cough and sputum or appearance of pulmonary rales in lung, sputum and appearance of pulmonary rales or wheezing rale in lung, dyspnea or extensive pulmonary rales or wheezing rale in lung.
7. Decreased level of consciousness includes presence of apathetic facial expressions, blurred mind, or coma.
8. Encephalopathy: an altered mental status that persisted for more than 24h, including lethargy, irritability, or a change in personality and behavior.
9. qSOFA score: quick sequential organ failure assessment. The qSOFA score includes respiratory rate ≥ 22/min, systolic blood pressure ≤ 100 mmHg, and abnormal mental status.
10. SIRS: systemic inflammatory response syndrome. SIRS criteria include respiratory rate > 20/min; temperature > 38℃ or < 36℃; pulse >90 beats/min; and white blood cell count > 12,000/μL or < 4000/μL.
11. MOD: multiple organ dysfunction was defined when two or more of the following conditions existed: (1) hypoxia requiring respirator-assisted ventilation for at least 3–5 days; (2) serum bilirubin≥2–3 mg/dL or liver function tests≥twice normal; (3) oliguria ≤479 mL/24h or rising creatinine (≥2–3 mg/dL); (4) ileus with intolerance to enteral feeding >5 days; (5) prothrombin time (PT) and PTT (partial thromboplastin time) increase >25% or platelet counts <50–80000; (6) confusion and mild disorientation; (7) decreased ejection fraction or capillary leak syndrome (Bone et al, 1992).
