# Supplementary material for: A model based on meta-analysis to evaluate poor prognosis of patients with severe fever with thrombocytopenia syndrome
Source: Front Microbiol. 2024 Jan 8;14:1307960. doi: 10.3389/fmicb.2023.1307960 (PMC10801726; doi:10.3389/fmicb.2023.1307960)
Supplement: Supplementary file 2 [file Data_Sheet_2.docx]

**Supplementary Table 1.** Baseline characteristics of the 24 cohorts included in the systematic review and meta-analysis.

| First Author/Year/  Country or region  (continent) | Study design  and period | Sample  Size (%female) | Age (years)^a^ | Risk factors |
| --- | --- | --- | --- | --- |
| Mutbyul Kim (5) /2022/Korea^1^ | Retrospective  2014.08~2021.09 | 53  （54.7%） | 68.4±12.6 | Age, Onset of illness to admission, Hemorrhagic manifestations^b^, MOD |
| Xiaowen Xu (7) /2018/China^2^ | Retrospective  2014.01~2015.12 | 60  （55.0%） | 65.82±11.36 | Neurological signs^c^, Hemorrhagic manifestations^b^, Disturbance of consciousness^d^, Serum sodium, CRP, APTT, Scr |
| Li Song (5) /2022/China^3^ | Retrospective  2015.01~2019.12 | 216  （45.8%） | 66.54±10.64 | Coma, Pulmonary infection, Viral load, APTT |
| Lianzi Wang (6) /2022/China^4^ | Retrospective  2019.09~2020.12 | 107  （54.5%） | 60.38±11.29 | PCT, AST/ALT-ratio |
| Feng He (3) /2021/China^5^ | Retrospective  2017.06~2021.01 | 100  （54%） | 65.20±9.86 | Disturbance of consciousness^d^, Hemorrhagic manifestations^b^, Kidney dysfunction, AST, LDH |
| Jianhua Hu (7) /2018/China^6^ | Retrospective  2014.01~2017.04 | 25  （40%） | 63 [26-74] | Lactate |
| Liu Z (11) /2022/China^7^ | Retrospective  2019.11~2021.11 | 194  （47.9%） | 62.39±11.85 | Age, EOS%, BAS%, AST, DBIL, EOS%+BAS% |
| Fang Qian (14) /2023/China^8^ | Retrospective  2011.05~2022.06 | 882  （51.5%） | 64 (56-71) | Age, APTT, CLR (CRP/LYM) |
| Zishuai Liu (11) /2022/China^9^ | Retrospective  2021.01~2021.12 | 155  （50.3%） | 61.98±11.70 | LYM, MON%, CK, ALP, ALB, CAR (CRP/ALB) |
| Yun Liu (5) /2022/China^10^ | Retrospective  2018.01~2021.06 | 182  （51.6%） | 59.24±12.74 | WBC, NEUT, NLR (NEUT/LYM) |
| **Supplementary Table 1.** Continued. | | | | |
| Yuanyuan Wei (12) /2022/China^11^ | Retrospective  2016.05~2019.10 | 228  （53.1%） | 63.0 (54.0-70.8) | NLR (NEUT/LYM), BUN, AST, AST/ALT-ratio |
| Xiankun Wang (22) /2022/China^12^ | Prospective  2011.04~2019.12 | 415  （41.9%） | 60.16±12.19 | UA, NLR (NEUT/LYM) |
| Xiaozhou Yang (5) /2022/China^13^ | Retrospective  2016.07~2021.11 | 82  （51.2%） | Survival 62 (55-71)  Fatal 67 (56.5-73.8) | MON, GGT, CAR (CRP/ALB) |
| Li Wang (6) /2017/China^14^ | Retrospective  2013.05~2015.11 | 174  （47.1%） | 63.38±10.41 | Age, AST, Scr |
| Zhipeng Zu (8) /2022/China^15^ | Retrospective  2011~2020 | 188  （45.7%） | 66.0 [33-88] | Age, Petechiae, Gingival haemorrhage, Melena |
| Heyon-Na Jo (23) /2022/China^16^ | Retrospective  2015~2018 | 56  （51.8%） | 66 (58-76) | Viral load, Initial APACHE II score^e^ |
| Bei Jia (15) /2017/China^17^ | Retrospective  2010.10~2017.07 | 142  （52.8%） | 58.3±12.0 | Age, BUN, APTT |
| Shue Xiong (9) /2016/Chian^18^ | Retrospective  2015.03-2015.11 | 179  （60.3%） | 58 [27-91] | Neurological symptom^f^, Respiratory symptom^g^, MON%, Viral load |
| Yonghui Gui (3) /2021/China^19^ | Prospective  2019.01~2021.06 | 127  （52.0%） | 60.36±10.91 | Age, BUN, PAR (PLT/ALB) |
| Wei Liu (19) /2013/China^20^ | Retrospective  2011~2012 | 311  （55.0%） | 61 [7-87] | Age, LDH, CK, Decreased level of consciousness^h^ |
| Fei Wang (5) /2020/China^21^ | Retrospective  2013.01~2019.01 | 51  （47.1%） | 61 [30-80] | Age, APTT |
| Lin Wang (25) /2019/China^22^ | Prospective  2011.04-2018.12 | 429  （46.9） | 60.8±12.1 | Age, Encephalopathy^i^, LYM%, LDH, BUN |
|  |  |  |  |  |
| **Supplementary Table 1.** Continued. | | | | |
| Qi Nie (9) /2020/China^23^ | Retrospective  2014.01~2019.12 | 116  （49.1%） | 63±9.1 | Age, creatinine, Lactate |
| Li Wang (4) /2020/China^24^ | Retrospective  2013.05~2017.07 | 321  （47.0%） | 63.8±11.2 | Age, AST, qSOFA score^j^, SIRS score^k^ |

1. Median (IQR) or Mean±SD or Median [range]
2. Haemorrhagic manifestations included skin ecchymosis, oral gingival bleeding, gastrointestinal bleeding, and pulmonary bleeding.
3. Neurological signs: abnormalities of the cranial nerves, abnormalities of motor system function (such as muscle wasting, muscle tone and power, posture and gait, involuntary movements, and ataxia), abnormalities of sensory function (hypoesthesia and paresthesia), abnormal neural reﬂexes (such as superﬁcial reﬂex, deep reﬂex, pathological reﬂex, and signs of meningeal irritation), and abnormalities of the autonomic system.
4. Disturbance of consciousness: drowsiness, confusion, lethargy, or a severe disturbance of consciousness (no Glasgow Coma Score evaluation was done).
5. APACHE II: Acute Physiology and Chronic Health Evaluation II.
6. Neurologic symptoms: limb tremor/blurred mind/slower reaction, strong decrease consist of drowsiness or frequent muscle convulsion, serious decrease of coma.
7. Respiratory symptoms: cough and sputum or appearance of pulmonary rales in lung, sputum and appearance of pulmonary rales or wheezing rale in lung, dyspnea or extensive pulmonary rales or wheezing rale in lung.
8. Decreased level of consciousness includes presence of apathetic facial expressions, blurred mind, or coma.
9. Encephalopathy: an altered mental status that persisted for more than 24h, including lethargy, irritability, or a change in personality and behavior.
10. qSOFA score: quick sequential organ failure assessment. The qSOFA score includes respiratory rate ≥ 22/min, systolic blood pressure ≤ 100 mmHg, and abnormal mental status.
11. SIRS: systemic inflammatory response syndrome. SIRS criteria include respiratory rate > 20/min; temperature > 38℃ or < 36℃; pulse >90 beats/min; and white blood cell count > 12,000/μL or < 4000/μL.
12. MOD: multiple organ dysfunction was defined when two or more of the following conditions existed: (1) hypoxia requiring respirator-assisted ventilation for at least 3–5 days; (2) serum bilirubin ≥2–3 mg/dL or liver function tests ≥twice normal; (3) oliguria ≤479 mL/24h or rising creatinine (≥2–3 mg/dL); (4) ileus with intolerance to enteral feeding >5 days; (5) prothrombin time (PT) and PTT (partial thromboplastin time) increase >25% or platelet counts <50–80000; (6) confusion and mild disorientation; (7) decreased ejection fraction or capillary leak syndrome (Bone et al, 1992).
13. Abbreviated word: CRP, C-reactive protein; PCT, procalcitonin; WBC, white blood cell; NEUT, neutrophile granulocyte; LYM, lymphocyte; EOS, eosinophilic granulocyte; BAS, basophilic granulocyte; MON, monocytes; PLT, thrombocyte; ALB, albumin; ALT, alanine aminotransferase; AST, aspertate aminotransferase; DBIL, direct bilirubin; ALP, alkaline phosphatase; GGT, gamma-glutamyltransferase; LDH, lactate dehydrogenase; CK, creatine kinase; APTT, activated partial thromboplastin time; BUN, blood urea nitrogen; Scr, serum creatinine; UA, uric acid; NLR, the ratio of neutrophil to lymphocyte; CAR, the ratio of C-reactive protein to albumin; PAR, the ratio of platelet to albumin; CLR, the ratio of C-reactive protein to lymphocyte.

**Supplementary Table 2.** Newcastle-Ottawa Quality Assessment Scale of the 24 cohort studies.

| Study (First author/year) | Study Population | Represen-  tativeness of the sample | Ascertain-ment of specimen collection methods | Sample size | Non-  respondents | Impact of Bias (selection bias, measurement bias, participant reporting, confounders) | Assessment of the outcome | Statistical analysis | Total Score |
| --- | --- | --- | --- | --- | --- | --- | --- | --- | --- |
| Mutbyul Kim (2022)^1^ | 1 | 2 | 1 | 0 | 0 | 1 | 1 | 1 | 7 |
| Xiaowen Xu (2018)^2^ | 1 | 2 | 0 | 0 | 0 | 1 | 1 | 1 | 6 |
| Li Song (2022)^3^ | 1 | 2 | 1 | 0 | 0 | 0 | 1 | 1 | 6 |
| Lianzi Wang (2022)^4^ | 1 | 2 | 1 | 0 | 0 | 1 | 1 | 1 | 7 |
| Feng He (2021)^5^ | 1 | 2 | 1 | 0 | 0 | 0 | 1 | 1 | 6 |
| Jianhua Hu (2018)^6^ | 1 | 2 | 1 | 0 | 0 | 1 | 1 | 1 | 7 |
| Liu Z (2022)^7^ | 1 | 2 | 1 | 0 | 0 | 1 | 1 | 1 | 7 |
| Fang Qian (2023)^8^ | 1 | 2 | 1 | 1 | 0 | 1 | 1 | 1 | 8 |
| Zishuai Liu (2022)^9^ | 1 | 2 | 1 | 0 | 0 | 1 | 1 | 1 | 7 |
| Yun Liu (2022)^10^ | 1 | 2 | 1 | 0 | 0 | 1 | 1 | 1 | 7 |
| Yuanyuan Wei (2022)^11^ | 1 | 2 | 1 | 0 | 0 | 0 | 1 | 1 | 6 |
| Xiankun Wang (2022)^12^ | 1 | 2 | 1 | 0 | 0 | 1 | 1 | 1 | 7 |
| Xiaozhou Yang (2022)^13^ | 1 | 2 | 1 | 0 | 0 | 1 | 1 | 1 | 7 |
| Li Wang (2017)^14^ | 1 | 2 | 1 | 0 | 0 | 1 | 1 | 1 | 7 |
|  |  |  |  |  |  |  |  |  |  |
| **Supplementary Table 2.** Continued. | | | | | | | | | |
| Zhipeng Zu (2022)^15^ | 1 | 2 | 1 | 0 | 0 | 1 | 1 | 1 | 7 |
| Heyon-Na Jo (2022)^16^ | 1 | 2 | 1 | 0 | 0 | 1 | 1 | 1 | 7 |
| Bei Jia (2017)^17^ | 1 | 2 | 1 | 0 | 0 | 1 | 1 | 1 | 7 |
| Shue Xiong (2016)^18^ | 1 | 2 | 1 | 0 | 0 | 1 | 1 | 1 | 7 |
| Yonghui Gui (2021)^19^ | 1 | 2 | 1 | 0 | 0 | 1 | 1 | 1 | 7 |
| Wei Liu (2013)^20^ | 1 | 2 | 1 | 0 | 0 | 1 | 1 | 1 | 7 |
| Fei Wang (2020)^21^ | 1 | 2 | 0 | 0 | 0 | 1 | 1 | 1 | 6 |
| Lin Wang (2019)^22^ | 1 | 2 | 1 | 0 | 0 | 1 | 1 | 1 | 7 |
| Qi Nie (2020)^23^ | 1 | 2 | 1 | 0 | 0 | 1 | 1 | 1 | 7 |
| Li Wang (2020)^24^ | 1 | 2 | 0 | 0 | 0 | 1 | 1 | 1 | 6 |

**Supplementary Table 3.** 49 risk factors included in the systematic review and meta-analysis.

| Risk factors | First author/Year | Sample size | Survival/Death | Definition of risk factor | RR | 95% CI | *P* value |
| --- | --- | --- | --- | --- | --- | --- | --- |
| Age | Mutbyul Kim/2022^1^ | 53 | 44/9 | Increment by 1 year | 1.14 | 1.05-1.23 | 0.001 |
|  | Liu Z/2022^7^ | 194 | 171/23 | Increment by 1 year | 1.070 | 1.007-1.137 | 0.028 |
|  | Fang Qian/2023^8^ | 882 | 725/157 | Increment by 1 year | 1.052 | 1.026-1.080 | ＜0.001 |
|  | Li Wang/2017^14^ | 174 | 134/40 | Increment by 1 year | 1.128 | 1.071-1.189 | ＜0.001 |
|  | Bei Jia/2017^17^ | 142 | 109/33 | Increment by 1 year | 1.117 | 1.046-1.194 | 0.001 |
|  | Wei Liu/2013^20^ | 311 | 257/54 | Increment by 1 year | 1.061 | 1.023-1.099 | 0.001 |
|  | Fei Wang/2020^21^ | 51 | 35/16 | Increment by 1 year | 1.245 | 1.052-1.474 | 0.011 |
|  | Li Wang/2020^24^ | 321 | 234/87 | Increment by 1 year | 1.107 | 1.039-1.176 | ＜0.0001 |
|  | Qi Nie/2020^23^ | 116 | 66/50 | Increment by 1 year | 1.109 | 1.027-1.198 | 0.009 |
|  | Lin Wang/2019^22^ | 429 | 360/69 | Increment by 1 year | 1.036 | 1.008-1.064 | 0.010 |
|  | Zhipeng Zu/2022^15^ | 188 | 148/40 | Age≥60 | 7.47 | 1.32-42.33 | 0.023 |
|  | Yonghui Gui/2021^19^ | 127 | 91/36 | Age≥65 | 0.208 | 0.069-0.63 | 0.005 |
| Onset of illness to admission | Mutbyul Kim /2022^1^ | 53 | 44/9 | Increment by 1 day | 0.48 | 0.29-0.8 | 0.005 |
| Hemorrhagic manifestations^a^ | Mutbyul Kim/2022^1^ | 53 | 44/9 | Hemorrhagic manifestations | 18.25 | 2.11-157.98 | 0.008 |
|  | Xiaowen Xu/2018^2^ | 60 | 40/20 | Hemorrhagic manifestations | 20.251 | 2.056-199.443 | 0.010 |
|  | Feng He/2021^5^ | 100 | 80/20 | Hemorrhagic manifestations | 19.367 | 5.820-64.454 | ＜0.001 |
| Petechiae | Zhipeng Zu/2022^15^ | 188 | 148/40 | Petechiae | 7.76 | 1.17-51.50 | 0.034 |
|  |  |  |  |  |  |  |  |
| **Supplementary Table 3.** Continued. | | | | | | | |
| Gingival haemorrhage | Zhipeng Zu/2022^15^ | 188 | 148/40 | Gingival haemorrhage | 5.38 | 1.25-23.15 | 0.024 |
| Melena | Zhipeng Zu/2022^15^ | 188 | 148/40 | Melena | 5.75 | 1.18-28.07 | 0.031 |
| Pulmonary infection | Li Song/2022^3^ | 216 | 162/54 | Pulmonary infection | 7.47 | 1.44-38.87 | 0.017 |
| Respiratory symptoms^b^ | Shue Xiong/2016^18^ | 179 | 145/34 | Respiratory symptoms | 4.480 | 1.654-12.134 | 0.003 |
| Kidney dysfunction | Feng He/2021^5^ | 100 | 80/20 | Kidney dysfunctiont | 32.824 | 6.923-155.624 | ＜0.001 |
| Encephalopathy^c^ | Lin Wang/2019^22^ | 429 | 360/69 | Encephalopathy | 13.028 | 6.771-25.068 | ＜0.001 |
| Coma | Li Song/2022^3^ | 216 | 162/54 | Coma | 35.23 | 6.42-193.45 | ＜0.001 |
| Disturbance of consciousness^d^ | Xiaowen Xu/2018^2^ | 60 | 40/20 | Disturbance of consciousness | 15.359 | 2.19-110.268 | 0.007 |
|  | Feng He/2021^5^ | 100 | 80/20 | Disturbance of consciousness | 4.086 | 1.454-11.483 | 0.008 |
| Decreased level of consciousness^e^ | Wei Liu/2013^20^ | 311 | 257/54 | Decreased  level of consciousness | 5.397 | 2.660-10.948 | ＜0.001 |
| Neurological signs^f^ | Xiaowen Xu/2018^2^ | 60 | 40/20 | Neurological signs | 31.247 | 4.813-202.853 | 0.000 |
| Neurologic symptoms^g^ | Shue Xiong/2016^18^ | 179 | 145/34 | Neurologic symptoms | 6.068 | 2.076-17.730 | 0.001 |
| qSOFA^h^ | Li Wang/2020^24^ | 321 | 234/87 | Increment by 1 | 3.654 | 1.488-6.378 | ＜0.0001 |
| SIRS^i^ | Li Wang/2020^24^ | 321 | 234/87 | Increment by 1 | 1.625 | 1.025-2.581 | 0.039 |
| MOD^j^ | Mutbyul Kim /2022^1^ | 53 | 44/9 | MOD | 2.36 | 1.04-5.38 | 0.041 |
| WBC | Yun Liu/2022^10^ | 182 | 158/24 | Increment by 1×10^9^/L | 1.263 | 1.120-1.424 | 0.001 |
| NEUT | Yun Liu/2022^10^ | 182 | 158/24 | Increment by 1×10^9^/L | 1.326 | 1.181-1.489 | 0.001 |
| EOS% | Liu Z/2022^7^ | 194 | 171/23 | Increment by 1% | 3.215 | 1.543-6.699 | 0.002 |
|  |  |  |  |  |  |  |  |
| **Supplementary Table 3.** Continued. | | | | | | | |
| BAS% | Liu Z/2022^7^ | 194 | 171/23 | Increment by 1% | 2.290 | 1.156-4.535 | 0.017 |
| NLR | Yun Liu/2022^10^ | 182 | 158/24 | Increment by 1 | 1.121 | 1.033-1.215 | 0.006 |
|  | Yuanyuan Wei/2022^11^ | 228 | 177/51 | Increment by 1 | 1.52 | 1.32-1.75 | 0.000 |
|  | Xiankun Wang/2022^12^ | 415 | 352/63 | NLR＞5.4 | 8.153 | 1.738-38.245 | 0.008 |
| EOS%+BAS% | Liu Z/2022^7^ | 194 | 171/23 | Increment by 1% | 282.867 | 19.263 – 4153.781 | 0.000 |
| MON | Xiaozhou Yang/2022^13^ | 82 | 50/32 | MON≤0.1×10^9^/L | 9.102 | 1.878-44.106 | 0.006 |
| LYM | Zishuai Liu/2022^9^ | 155 | 133/22 | Increment by 1×10^9^/L | 0.132 | 0.033-0.523 | 0.004 |
|  | Lin Wang/2019^22^ | 429 | 360/69 | Increment by 1×10^9^/L | 0.963 | 0.943-0.983 | ＜0.001 |
| Serum sodium | Xiaowen Xu/2018^2^ | 60 | 40/20 | Serum sodium＜136mmol/l | 5.280 | 1.235-22.575 | 0.025 |
| LDH | Feng He/2021^5^ | 100 | 80/20 | LDH≥1000U/L | 37.000 | 9.982-137.152 | ＜0.001 |
|  | Wei Liu/2013^20^ | 311 | 257/54 | LDH＞1200U/L  & Increment by 1U/L | 2.620 | 1.073-6.399 | 0.035 |
|  | Lin Wang/2019^22^ | 429 | 360/69 | Increment by 1ULN | 1.381 | 1.111-1.718 | 0.004 |
| CK | Zishuai Liu/2022^9^ | 155 | 133/22 | Increment by 1U/L | 1.001 | 1.000-1.001 | 0.000 |
|  | Wei Liu/2013^20^ | 311 | 257/54 | LDH＞800U/L  & Increment by 1U/L | 2.328 | 1.129-4.800 | 0..022 |
| AST | Feng He/2021^5^ | 100 | 80/20 | AST≥400U/L | 15.00 | 3.250-69.230 | ＜0.001 |
|  | Lin Z/2022^7^ | 194 | 171/23 | Increment by 1U/L | 1.003 | 1.001-1.005 | 0.001 |
|  | Yuanyuan Wei/2022^11^ | 228 | 117/51 | Increment by 1U/L | 1.00 | 1.00-1.00 | 0.029 |
|  | Li Wang/2017^14^ | 174 | 134/40 | Increment by 1U/L | 1.002 | 1.001-1.003 | ＜0.001 |
|  | Li Wang/2020^24^ | 321 | 234/87 | Increment by 1U/L | 1.002 | 1.000-1.003 | 0.01 |
|  |  |  |  |  |  |  |  |
| **Supplementary Table 3.** Continued. | | | | | | | |
| AST/ALT-ratio | Lianzi Wang/2022^4^ | 107 | 81/26 | AST/ALT ＞2.33 | 3.38 | 1.22-9.30 | 0.018 |
|  | Yuanyuan Wei/2022^11^ | 228 | 177/51 | Increment by 1 | 1.34 | 1.13-1.60 | 0.001 |
| ALB | Zishuai Liu/2022^9^ | 155 | 133/22 | Increment by 1g/L | 0.805 | 0.722-0.897 | 0.000 |
| DBIL | Liu Z/2022^7^ | 194 | 171/23 | Increment by 1μmol/L | 1.120 | 1.004-1.248 | 0.041 |
| ALP | Zishuai Liu/2022^9^ | 155 | 133/22 | Increment by 1U/L | 1.006 | 1.002-1.011 | 0.006 |
| GGT | Xiaozhou Yang/2022^13^ | 82 | 50/32 | GGT≥2ULN | 4.643 | 1.003-21.491 | 0.05 |
| PCT | Lianzi Wang/2022^4^ | 107 | 81/26 | PCT＞0.5ng/ml | 2.55 | 1.11-5.19 | 0.034 |
| CRP | Xiaowen Xu/2018^2^ | 60 | 40/20 | CRP≥20mg/l | 2.641 | 1.090-6.396 | 0..031 |
| CLR | Fang Qian/2023^8^ | 882 | 725/157 | Increment by 1 | 1.053 | 1.033-1.073 | ＜0.001 |
| CAR | Zishuai Liu/2022^9^ | 155 | 133/22 | Increment by 1 | 2.585 | 1.405-4.753 | 0.002 |
|  | Xiaozhou Yang/2022^13^ | 82 | 50/32 | CAR≥0.14 | 4.801 | 1.049-21.977 | 0.043 |
| PAR | Yonghui Gui/2021^19^ | 127 | 91/36 | PAR＞1.43 | 4.023 | 1.204-13.436 | 0.024 |
| APTT | Xiaowen Xu/2018^2^ | 60 | 40/20 | APTT≥40s | 6.018 | 1.450-24.975 | 0.013 |
|  | Li Song/2022^3^ | 216 | 162/54 | APTT≥40s | 33.02 | 1.49-733.16 | 0.027 |
|  | Fang Qian/2023^8^ | 882 | 725/157 | Increment by 1s | 1.061 | 1.033-1.090 | ＜0.001 |
|  | Bei Jia/2017^17^ | 142 | 109/33 | Increment by 1s | 1.093 | 1.041-1.148 | ＜0.0001 |
|  | Fei Wang/2020^21^ | 51 | 35/16 | Increment by 1s | 1.095 | 1.005-1.192 | 0.038 |
| BUN | Jianhua Hu/2018^6^ | 25 | 20/5 | BUN＞8.2μmol/l | 76.0 | 3.883-1487.520 | 0.004 |
|  | Yonghui Gui/2021^19^ | 127 | 91/36 | BUN＞7mmol/l | 0.236 | 0.074-0.751 | 0.014 |
|  | Yuanyuan Wei/2022^11^ | 228 | 177/51 | Increment by 1mmol/L | 1.11 | 1.05-1.18 | 0.001 |
|  | Bei Jia/2017^17^ | 142 | 109/33 | Increment by 1mmol/L | 1.277 | 1.084-1.505 | 0.003 |
|  | Lin Wang/2019^22^ | 429 | 360/69 | Increment by 1mmol/l | 1.055 | 1.026-1.084 | ＜0.001 |
| UA | Xiankun Wang/2022^12^ | 415 | 352/63 | UA＞420μmol/l | 14.005 | 3.002-65.336 | 0.001 |
|  |  |  |  |  |  |  |  |
| **Supplementary Table 3.** Continued. | | | | | | | |
| Scr | Xiaowen Xu/2018^2^ | 60 | 40/20 | Scr≥130μmol/l | 6.776 | 1.047-43.840 | 0.045 |
|  | Li Wang/2017^14^ | 174 | 134/40 | Increment by 1μmol/l | 1.013 | 1.006-1.020 | ＜0.001 |
| Creatinine | Qi Nie/2020^23^ | 116 | 66/50 | Increment by 1μmol/L | 1.022 | 1.004-1.041 | 0.019 |
| Lactate | Qi Nie/2020^23^ | 116 | 66/50 | Increment by 1mmol/L | 1.554 | 1.018-2.372 | 0.041 |
| Viral load | Li Song/2022^3^ | 216 | 162/54 | Increment by 1 copy/ml | 6.40 | 1.19-34.51 | 0.031 |
|  | Shue Xiong/2016^18^ | 179 | 145/34 | Increment by 1 copy/ml | 5.017 | 1.868-13.478 | 0.001 |
|  | Heyon-Na Jo/2022^16^ | 56 | 44/12 | Viral load≥10^4^copies/ml | 38.298 | 1.583-926.593 | 0.025 |
| .Initial APACHE II score^k^ | Heyon-Na Jo/2022^16^ | 56 | 44/12 | Increment by 1 | 1.306 | 1.031-1.655 | 0.027 |

1. Haemorrhagic manifestations included skin ecchymosis, oral gingival bleeding, gastrointestinal bleeding, and pulmonary bleeding.
2. Respiratory symptoms: cough and sputum or appearance of pulmonary rales in lung, sputum and appearance of pulmonary rales or wheezing rale in lung, dyspnea or extensive pulmonary rales or wheezing rale in lung.
3. Encephalopathy: an altered mental status that persisted for more than 24h, including lethargy, irritability, or a change in personality and behavior.
4. Disturbance of consciousness: drowsiness, confusion, lethargy, or a severe disturbance of consciousness (no Glasgow Coma Score evaluation was done).
5. Decreased level of consciousness includes presence of apathetic facial expressions, blurred mind, or coma.
6. Neurological signs: abnormalities of the cranial nerves, abnormalities of motor system function (such as muscle wasting, muscle tone and power, posture and gait, involuntary movements, and ataxia), abnormalities of sensory function (hypoesthesia and paresthesia), abnormal neural reﬂexes (such as superﬁcial reﬂex, deep reﬂex, pathological reﬂex, and signs of meningeal irritation), and abnormalities of the autonomic system.
7. Neurologic symptoms: limb tremor/blurred mind/slower reaction, strong decrease consist of drowsiness or frequent muscle convulsion, serious decrease of coma.
8. qSOFA: quick sequential organ failure assessment. The qSOFA score includes respiratory rate ≥ 22/min, systolic blood pressure ≤ 100 mmHg, and abnormal mental status.
9. SIRS: systemic inflammatory response syndrome. SIRS criteria include respiratory rate > 20/min; temperature > 38℃ or < 36℃; pulse >90 beats/min; and white blood cell count > 12,000/μL or < 4000/μL.
10. MOD: Multiple organ dysfunction was defined when two or more of the following conditions existed: (1) hypoxia requiring respirator-assisted ventilation for at least 3–5 days; (2) serum bilirubin ≥2–3 mg/dL or liver function tests≥twice normal; (3) oliguria≤479 mL/24h or rising creatinine (≥2–3 mg/dL); (4) ileus with intolerance to enteral feeding >5 days; (5) prothrombin time (PT) (PT) and PTT (partial thromboplastin time) increase >25% or platelet counts <50–80000; (6) confusion and mild disorientation; and (7) decreased ejection fraction or capillary leak syndrome (Bone et al, 1992).
11. APACHE II: Acute Physiology and Chronic Health Evaluation II.
12. Abbreviated word: CRP, C-reactive protein; PCT, procalcitonin; WBC, white blood cell; NEUT, neutrophile granulocyte; LYM, lymphocyte; EOS, eosinophilic granulocyte; BAS, basophilic granulocyte; MON, monocytes; PLT, thrombocyte; ALB, albumin; ALT, alanine aminotransferase; AST, aspertate aminotransferase; DBIL, direct bilirubin; ALP, alkaline phosphatase; GGT, gamma-glutamyltransferase; LDH, lactate dehydrogenase; CK, creatine kinase; APTT, activated partial thromboplastin time; BUN, blood urea nitrogen; Scr, serum creatinine; UA, uric acid; NLR, the ratio of neutrophil to lymphocyte; CAR, the ratio of C-reactive protein to albumin; PAR, the ratio of platelet to albumin; CLR, the ratio of C-reactive protein to lymphocyte.

**Supplementary Table 4.** Baseline characteristics of patients in validation cohort.

| Parameters [Normal range] | Total  （n=194） | Survival  （n=171） | Deceased  （n=23） | *P* value |
| --- | --- | --- | --- | --- |
| **Age (years)** | 62.39 ± 11.85 | 61.20 ± 11.38 | 71.22 ± 11.76 | ＜0.001 |
| ≤45 [n (%)] | 16 (8.2) | 15 (8.8) | 1 (4.3) | 0.749 |
| 46-60 [n (%)] | 64 (33.0) | 62 (36.3) | 2 (8.7) | 0.008 |
| 61-75 [n (%)] | 86 (44.3) | 75 (43.9) | 11 (47.8) | 0.719 |
| ≥75 [n (%)] | 28 (14.4) | 19 (11.1) | 9 (39.1) | 0.001 |
| **Female [n (%)]** | 93 (47.94) | 82 (47.95) | 11 (47.83) | 0.991 |
| **Time from onset to admission (days)** | 5.0 (4.0–7.0) | 5.0 (4.0–7.0) | 5.0 (4.0–7.0) | 0.954 |
| **Hospitalization (days)** | 10.0 (6.0–13.0) | 10.0 (7.0–13.0) | 4.0 (2.0–6.0) | ＜0.001 |
| **History [n (%)]** |  |  |  |  |
| CHD | 11/194 (5.7) | 9/171 (5.3) | 2/23 (8.7) | 0.851 |
| Hypertensive disease | 31/194 (16.0) | 28/171 (16.4) | 3/23 (13.0) | 0.915 |
| Liver disease | 6/194 (3.1) | 5/171 (2.9) | 1/23 (4.3) | 0.536 |
| Kidney disease | 1/194 (0.5) | 1/171 (0.6) | 0 | 1.000 |
| Diabetes | 7/194 (3.6) | 7/171 (4.1) | 0 | 1.000 |
| Tumor related history | 4/194 (2.1) | 4/171 (2.3) | 0 | 1.000 |
| **Symptoms and signs [n (%)]** |  |  |  |  |
| Fever [36.3-37.2] | 127 (65.5) | 112 (65.5) | 15 (65.2) | 0.545 |
| **Supplementary Table 4.** Continued. | | | | |
| Arthralgia | 81/194 (41.8) | 73/171 (42.7) | 8/23 (34.8) | 0.470 |
| Neurological Symptoms | 23 (11.9) | 15 (8.8) | 8 (34.8) | 0.001 |
| Hemorrhage | 6 (3.1) | 2 (1.2) | 4 (17.4) | 0.002 |
| **Blood routine** |  |  |  |  |
| WBC [3.5-9.5] (×10^9^/L) | 2.39 (1.50–4.06) | 2.38 (1.51–3.86) | 2.85 (1.36–4.25) | 0.662 |
| NEUT [1.8-6.3] (×10^9^/L) | 1.31 (0.80–2.48) | 1.24 (0.79–2.44) | 2.02 (0.91–3.34) | 0.293 |
| NEUT% [40-75] (%) | 61.85 (46.94–74.46) | 59.70 (46.94–73.74) | 69.90 (44.80–77.90) | 0.363 |
| LYM [1.1-3.2] (×10^9^/L) | 0.62 (0.38–1.15) | 0.63 (0.39–1.21) | 0.44 (0.31–0.80) | 0.079 |
| LYM% [20-50] (%) | 29.62 (16.99–42.64) | 30.30 (19.20–43.54) | 19.40 (14.80–33.80) | 0.017 |
| MON [0.1-0.6] (×10^9^/L) | 0.15 (0.07–0.36) | 0.15 (0.07–0.33) | 0.16 (0.03–0.56) | 0.682 |
| MON% [3-10] (%) | 6.50 (3.64–10.47) | 6.50 (3.80–9.84) | 7.40 (1.60–27.00) | 0.669 |
| RBC [3.8-5.1] (×10^9^/L) | 4.49 (4.14–4.86) | 4.48 (4.16–4.86) | 4.57 (3.76–5.04) | 0.584 |
| HBG [115-150] (g/L) | 137.50 (127.00–149.00) | 137.00 (127.00–147.00) | 148.00 (126.00–155.00) | 0.198 |
| PLT [125-350] (×10^9^/L) | 57.50 (40.00–78.75) | 59.00 (41.00–84.00) | 39.00 (18.00–59.00) | ＜0.001 |
| MPV [6.5-12] (fL) | 11.20 (10.28–12.00) | 11.30 (10.50–12.20) | 10.0 (8.60–11.00) | ＜0.001 |
| **Biochemical marker** |  |  |  |  |
| CK [0-167] (U/L) | 374.5 (170.00–997.00) | 326.0 (145.00–890.12) | 1655.9 (2663.00–425.90) | ＜0.001 |
| CKMB [0-24] (U/L) | 23.73 (14.95, 42.00) | 21.00 (14.61, 35.51) | 59.80 (32.00, 76.00) | < 0.001 |
| **Supplementary Table 4.** Continued. | | | | |
| LDH [109-245] (U/L) | 668.0 (392.00–1076.50) | 573.4 (375.00–922.00) | 900.0 (682.00–2506.35) | ＜0.001 |
| ALT [0-40] (U/L) | 89.00 (49.00,165.56) | 80.53 (47.00, 136.00) | 165.10 (109.00, 283.00) | < 0.001 |
| AST [0-40] (U/L) | 160.15 (80.33, 323.93) | 153.00 (71.00, 267.00) | 645.00 (247.00, 1082.00) | < 0.001 |
| TBIL [3.4-17.1] (μmol/L) | 10.20 (7.79, 14.03) | 10.10 (7.40, 13.50) | 15.10 (9.00, 23.70) | 0.002 |
| DBIL [0-6.8] (μmol/L) | 4.65 (3.40, 6.30) | 4.50 (3.20, 6.08) | 6.89 (5.06, 12.42) | < 0.001 |
| ALB [35-53] (g/L) | 33.37 (30.00, 36.62) | 33.70 (31.00,37.00) | 28.10 (27.00, 31.00) | < 0.001 |
| GLOB [20-40] (g/L) | 24.47 (21.68, 26.80) | 24.41 (21.50, 26.60) | 24.70 (22.55, 28.20) | 0.193 |
| GGT [7-32] (U/L) | 50.50 (24.00, 96.00) | 46.00 (23.00, 96.00) | 113.00 (65.00, 195.80) | < 0.001 |
| ALP [40-150] (U/L) | 67.00 (52.76, 87.75) | 65.00 (52.00, 80.00) | 96.00 (66.00, 192.00) | < 0.001 |
| BUN [3.2-7.1] (mmol/L) | 5.28 (4.10, 7.16) | 5.09 (4.07, 7.13) | 7.34 (5.97, 11.13) | < 0.001 |
| Scr [44-133] (μmol/L) | 66.28 (54.45, 81.27) | 64.20 (54.00, 81.00) | 81.27 (63.50, 110.90) | 0.002 |
| Glu [4.16-6.44] (mmol/L) | 6.57 (5.60, 8.16) | 6.51 (5.58, 8.16) | 7.50 (5.70, 9.00) | 0.317 |
| K^+^ [3.5-5.5] (mmol/L) | 3.84 (3.53–4.15) | 3.81 (3.53–4.15) | 3.90 (3.67–4.15) | 0.629 |
| Na^+^ [135-145] (mmol/L) | 134.00 (130.80, 136.60) | 134.00 (130.40, 136.70) | 134.00 (131.40, 136.00) | 0.825 |
| Ca^2+^ [2.25-2.75] (mmol/L) | 1.95 (1.90–2.02) | 1.95 (1.92–2.02) | 1.91 (1.76–1.97) | 0.009 |
| PCT [0-0.5] (ng/L) | 0.60 (0.12, 0.81) | 0.46 (0.11, 0.81) | 0.81 (0.50, 1.72) | 0.001 |
| **Supplementary Table 4.** Continued. | | | | |
| CRP [0-8] (mg/L) | 7.69 (2.30, 11.64) | 6.10 (1.98, 11.64) | 11.64 (10.20, 23.70) | 0.001 |
| **Coagulation** |  |  |  |  |
| TT [14-21] (s) | 19.65 (17.20, 21.00) | 19.65 (17.50, 20.90) | 18.80 (15.80, 24.90) | 0.763 |
| INR [0.8-1.5] | 1.02 (1.00, 1.09) | 1.02 (1.00, 1.09) | 1.01 (0.95, 1.10) | 0.372 |
| APTT [24-36] (s) | 46.90 (38.25, 48.61) | 46.70 (38.60, 48.61) | 61.30 (33.70, 85.00) | 0.052 |
| PT [11-14.5] (s) | 13.00 (11.98, 14.74) | 13.20 (12.00, 14.74) | 12.60 (11.50, 13.80) | 0.177 |
| **Model Scores’** | 54.50 (41.75, 70.00) | 54.00 (39.00, 68.00) | 72.00 (62.00, 100.00) | < 0.001 |

1. Median (interquartile ranges, IQR) or Mean ± SD; t test was used to compare the parametric variables of the survival and deceased groups, and Chi-square tests were used to compare non-parametric variables of the survival and deceased groups.
2. Abbreviated word: CHD, coronary atherosclerotic heart disease; WBC, white blood cell; NEUT, neutrophile granulocyte; LYM, lymphocyte; MON, monocytes; RBC, erythrocyte/red blood cell; HGB, haemoglobin; PLT, thrombocyte; MPV, mean platelet volume; CK, creatine kinase; CKMB, creatine kinase isoenzyme; LDH, lactate dehydrogenase; ALT, alanine aminotransferase; AST, aspertate aminotransferase; TBIL, total bilirubin; DBIL, direct bilirubin; ALB, albumin; GLOB, globulin; ALP, alkaline phosphatase; GGT, gamma-glutamyltransferase; BUN, blood urea nitrogen; Scr, serum creatinine; Glu, glucose; K+, kalium; Na+, natrium; Ca2+, calcium; PCT, procalcitonin; CRP, C-reactive protein; TT, thrombin time; INR, international normalized ratio; APTT, activated partial thromboplastin time; PT, prothrombin time.

**Supplementary Table 5.** No. of revolving studies, sample size, pooled RRs (95% CIs) and β-coefficient of risk factors included in the SFTS risk prediction model.

| Risk factors | No. of studies | Sample size | Pooled RR | 95% CI | β- coefficient |
| --- | --- | --- | --- | --- | --- |
| Age (by 1 years) | 10 | 2673 | 1.08 | 1.06-1.11 | 0.08 |
| Hemorrhagic manifestations | 4 | 401 | 10.63 | 5.46-20.72 | 2.36 |
| Encephalopathy | 5 | 1116 | 9.00 | 4.64-17.43 | 2.20 |
| APTT(by 1s) | 3 | 1075 | 1.07 | 1.05-1.09 | 0.07 |
| Scr(by 1μmol/L) | 2 | 290 | 1.01 | 1.01-1.02 | 0.01 |
| BUN(by 1mmol/L) | 3 | 799 | 1.10 | 1.03-1.18 | 0.10 |
| Viral Load | 3 | 451 | 6.09 | 2.67-13.87 | 1.81 |
| NLR(by 1) | 3 | 825 | 1.41 | 1.00-1.98 | 0.34 |
| CAR(by 1) | 2 | 237 | 2.82 | 1.60-4.96 | 1.04 |

Abbreviated word: BUN, blood urea nitrogen; Scr, serum creatinine; APTT, activated partial thromboplastin time; NLR, the ratio of neutrophil to lymphocyte; CAR, the ratio of C-reactive protein to albumin.

**Supplementary Table 6.** Multivariate logistic regression analysis of prognostic risk factors in patients with SFTS.

| **Risk factors** | **OR** | **95% CI** | ***P* value** |
| --- | --- | --- | --- |
| Model Scores | 1.032 | 1.002-1.063 | 0.034 |
| CKMB | 1.010 | 1.001-1.020 | 0.037 |
| AST | 1.003 | 1.001-1.005 | ＜0.001 |
| CRP | 1.052 | 1.004-1.102 | 0.035 |

**Supplementary Table 7.** Multivariate logistic regression analysis of the mortality risk prediction model for SFTS patients.

|  | OR | 95% CI | P value |
| --- | --- | --- | --- |
| Step 1^a^ |  |  |  |
| Model Scores | 1.056 | 1.032-1.081 | ＜0.001 |
| Step 2^b^ |  |  |  |
| Model Scores | 1.056 | 1.032-1.081 | ＜0.001 |
| Step 3^c^ |  |  |  |
| Model Scores | 1.049 | 1.024-1.075 | ＜0.001 |
| Step 4^d^ |  |  |  |
| Model Scores | 1.050 | 1.024-1.077 | ＜0.001 |

1. Step 1: unadjusted.
2. Step 2: adjusted for gender.
3. Step 3: adjusted gender, temperature, arthralgia, digestive symptoms and neurological signs.
4. Step 4: adjusting for gender, temperature, arthralgia, hemorrhage, digestive symptoms, neurological signs, hypertensive disease, diabetes, Coronary Heart Disease, chronic liver disease, chronic renal disease and tumor.

**Reference**

1. Kim M, Hong KW, Kim SC, Kim RB, Cho MC. Analysis of Clinical Characteristics and Laboratory Data Related to the Prognosis of Korean Severe Fever with Thrombocytopenia Syndrome Patients: A Single-Center Study. Vector Borne Zoonotic Dis. 2022 Nov;22(11):559-567. doi: 10.1089/vbz.2022.0035.
2. Xu X, Sun Z, Liu J, Zhang J, Liu T, Mu X, et al. Analysis of clinical features and early warning indicators of death from severe fever with thrombocytopenia syndrome. Int J Infect Dis. 2018 Aug;73:43-48. doi: 10.1016/j.ijid.2018.05.013.
3. Song L, Zhao Y, Wang G, Huang D, Sai L. Analysis of risk factors associated with fatal outcome among severe fever with thrombocytopenia syndrome patients from 2015 to 2019 in Shandong, China. Eur J Clin Microbiol Infect Dis. 2022 Dec;41(12):1415-1420. doi: 10.1007/s10096-022-04506-4.
4. Wang L, Xu Y, Zhang S, Bibi A, Xu Y, Li T. The AST/ALT Ratio (De Ritis Ratio) Represents an Unfavorable Prognosis in Patients in Early-Stage SFTS: An Observational Cohort Study. Front Cell Infect Microbiol. 2022 Feb 8;12:7256e42. doi: 10.3389/fcimb.2022.725642.
5. He F, Zheng X, Zhang Z. Clinical features of severe fever with thrombocytopenia syndrome and analysis of risk factors for mortality. BMC Infect Dis. 2021 Dec 14;21(1):1253. doi: 10.1186/s12879-021-06946-3.
6. Hu J, Li S, Zhang X, Zhao H, Yang M, Xu L, et al. Correlations between clinical features and death in patients with severe fever with thrombocytopenia syndrome. Medicine (Baltimore). 2018 Jun;97(22):e10848. doi: 10.1097/MD.0000000000010848.
7. Liu Z, Zhang R, Liu Y, Ma R, Zhang L, Zhao Z, et al. Eosinophils and basophils in severe fever with thrombocytopenia syndrome patients: Risk factors for predicting the prognosis on admission. PLoS Negl Trop Dis. 2022 Dec 21;16(12):e0010967. doi: 10.1371/journal.pntd.0010967.
8. Qian F, Zhou W, Liu Y, Ge Z, Lai J, Zhao Z, et al. High C-reactive protein to lymphocyte ratio predicts mortality outcomes of patients with severe fever with thrombocytopenia syndrome: A multicenter study in China. J Med Virol. 2023 Feb;95(2):e28546. doi: 10.1002/jmv.28546.
9. Liu Z, Zhang R, Zhou W, Ma R, Han L, Zhao Z, et al. High levels of C-reactive protein-to-albumin ratio (CAR) are associated with a poor prognosis in patients with severe fever with thrombocytopenia syndrome in early stage. J Med Virol. 2022 Nov;94(11):5375-5384. doi: 10.1002/jmv.27972.
10. Liu Y, Ni J, Xiong Y, Wu C, He F. Neutrophil-to-lymphocyte ratio is associated with 28-day mortality in patients with severe fever with thrombocytopenia syndrome. BMC Infect Dis. 2022 Mar 6;22(1):225. doi: 10.1186/s12879-022-07206-8.
11. Wei Y, Wang Z, Kang L, He L, Sheng N, Qin J, et al. NLR, A Convenient Early-Warning Biomarker of Fatal Outcome in Patients With Severe Fever With Thrombocytopenia Syndrome. Front Microbiol. 2022 Jun 23;13:907888. doi: 10.3389/fmicb.2022.907888.
12. Wang X, Lin L, Zhao Z, Zhou W, Ge Z, Shen Y, et al. The predictive effect of the platelet-to-lymphocyte ratio (PLR) and the neutrophil-to-lymphocyte ratio (NLR) on the risk of death in patients with severe fever with thrombocytopenia syndrome (SFTS): a multi-center study in China. Ann Transl Med. 2021 Feb;9(3):208. doi: 10.21037/atm-20-4736.
13. Yang X, Yin H, Xiao C, Li R, Liu Y. The Prognostic Significance of C-Reactive Protein to Albumin Ratio in Patients With Severe Fever With Thrombocytopenia Syndrome. Front Med (Lausanne). 2022 Apr 29;9:879982. doi: 10.3389/fmed.2022.879982.
14. Wang L, Zou Z, Hou C, Liu X, Jiang F, Yu H. Score risk model for predicting severe fever with thrombocytopenia syndrome mortality. BMC Infect Dis. 2017 Jan 7;17(1):42. doi: 10.1186/s12879-016-2111-0.
15. Zu Z, Hu Y, Zheng X, Chen C, Zhao Y, Jin Y, et al. A ten-year assessment of the epidemiological features and fatal risk factors of hospitalised severe fever with thrombocytopenia syndrome in Eastern China. Epidemiol Infect. 2022 Jun 21;150:e131. doi: 10.1017/S0950268822001108.
16. Jo HN, Kim J, Hwang SY, Seo JW, Kim DY, Yun NR, et al. Viral Load as a Factor Affecting the Fatality of Patients Suffering from Severe Fever with Thrombocytopenia Syndrome. Viruses. 2022 Apr 23;14(5):881. doi: 10.3390/v14050881.
17. Jia B, Yan X, Chen Y, Wang G, Liu Y, Xu B, et al. A scoring model for predicting prognosis of patients with severe fever with thrombocytopenia syndrome. PLoS Negl Trop Dis. 2017 Sep 21;11(9):e0005909. doi: 10.1371/journal.pntd.0005909.
18. Xiong S, Zhang W, Li M, Xiong Y, Li M, Wang H, et al. A simple and practical score model for predicting the mortality of severe fever with thrombocytopenia syndrome patients. Medicine (Baltimore). 2016 Dec;95(52):e5708. doi: 10.1097/MD.0000000000005708.
19. Gui Y, Xu Y, Yang P. Predictive Value of the Platelet-to-Albumin Ratio (PAR) on the Risk of Death at Admission in Patients Suffering from Severe Fever with Thrombocytopenia Syndrome. J Inflamm Res. 2021 Oct 29;14:5647-5652. doi: 10.2147/JIR.S335727.
20. Liu W, Lu QB, Cui N, Li H, Wang LY, Liu K, et al. Case-fatality ratio and effectiveness of ribavirin therapy among hospitalized patients in china who had severe fever with thrombocytopenia syndrome. Clin Infect Dis. 2013 Nov;57(9):1292-9. doi: 10.1093/cid/cit530.
21. Wang F, Wu Y, Jiao J, Wang J, Ge Z. Risk Factors and Clinical Characteristics of Severe Fever with Thrombocytopenia Syndrome. Int J Gen Med. 2020 Dec 30;13:1661-1667. doi: 10.2147/IJGM.S292735.
22. Wang L, Wan G, Shen Y, Zhao Z, Lin L, Zhang W, et al. A nomogram to predict mortality in patients with severe fever with thrombocytopenia syndrome at the early stage-A multicenter study in China. PLoS Negl Trop Dis. 2019 Nov 25;13(11):e0007829. doi: 10.1371/journal.pntd.0007829.
23. Nie Q, Wang D, Ning Z, Li T, Tian X, Bian P, et al. Analysis of Severe Fever With Thrombocytopenia Syndrome in Critical Ill Patients in Central China. Shock. 2020 Oct;54(4):451-457. doi: 10.1097/SHK.0000000000001527.
24. Wang L, Zou Z, Ding K, Hou C. Predictive risk score model for severe fever with thrombocytopenia syndrome mortality based on qSOFA and SIRS scoring system. BMC Infect Dis. 2020 Aug 12;20(1):595. doi: 10.1186/s12879-020-05299-7.
