# Supplementary material for: A model based on meta-analysis to evaluate poor prognosis of patients with severe fever with thrombocytopenia syndrome
Source: Front Microbiol. 2024 Jan 8;14:1307960. doi: 10.3389/fmicb.2023.1307960 (PMC10801726; doi:10.3389/fmicb.2023.1307960)
Supplement: Supplementary file 3 [file Data_Sheet_3.docx]

**Supplementary Figure 1.** Age

A


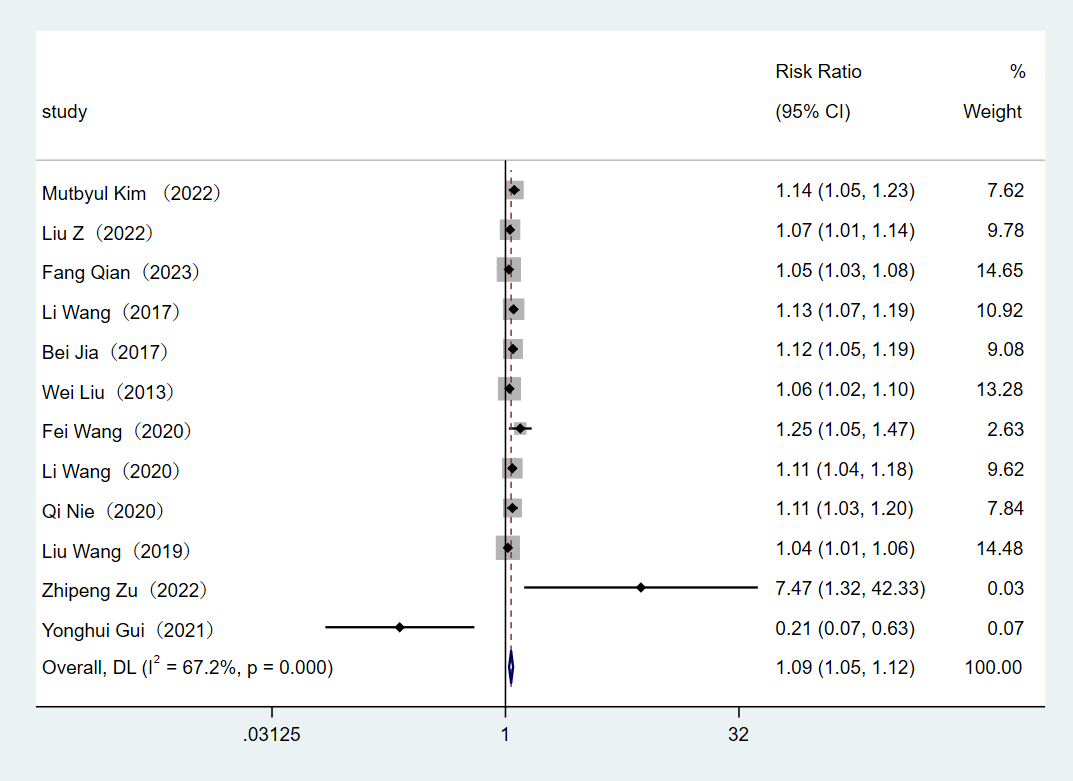


B


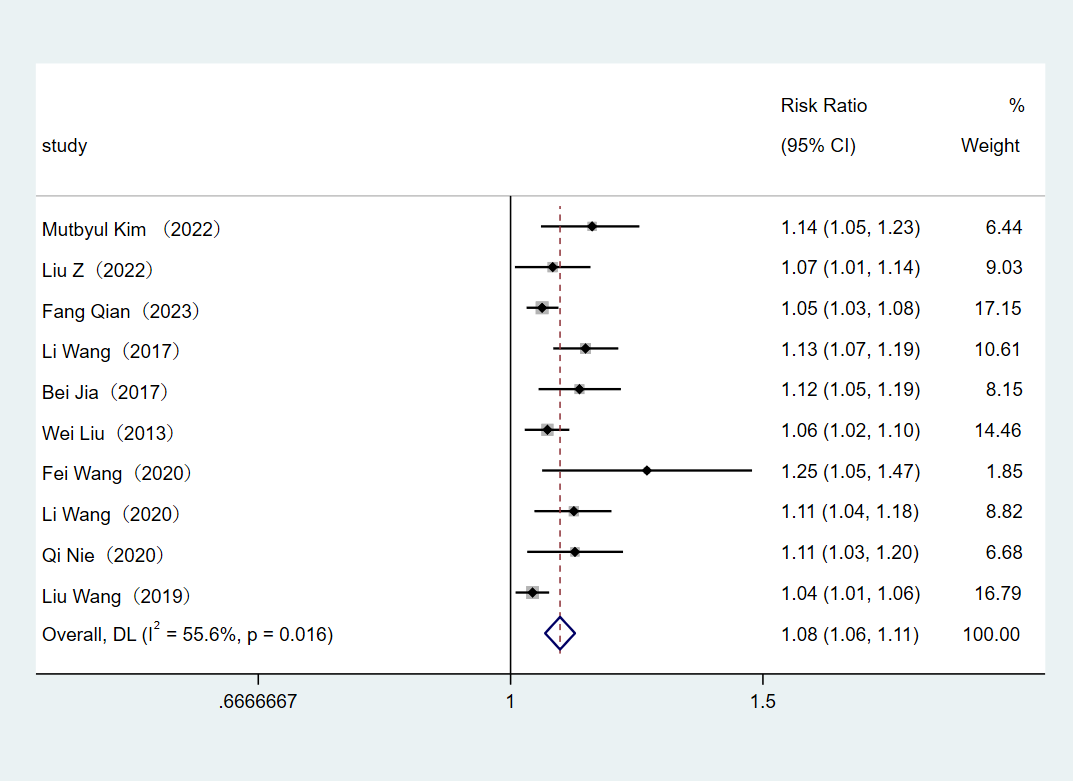


**Supplementary Figure 2.** Hemorrhagic manifestations


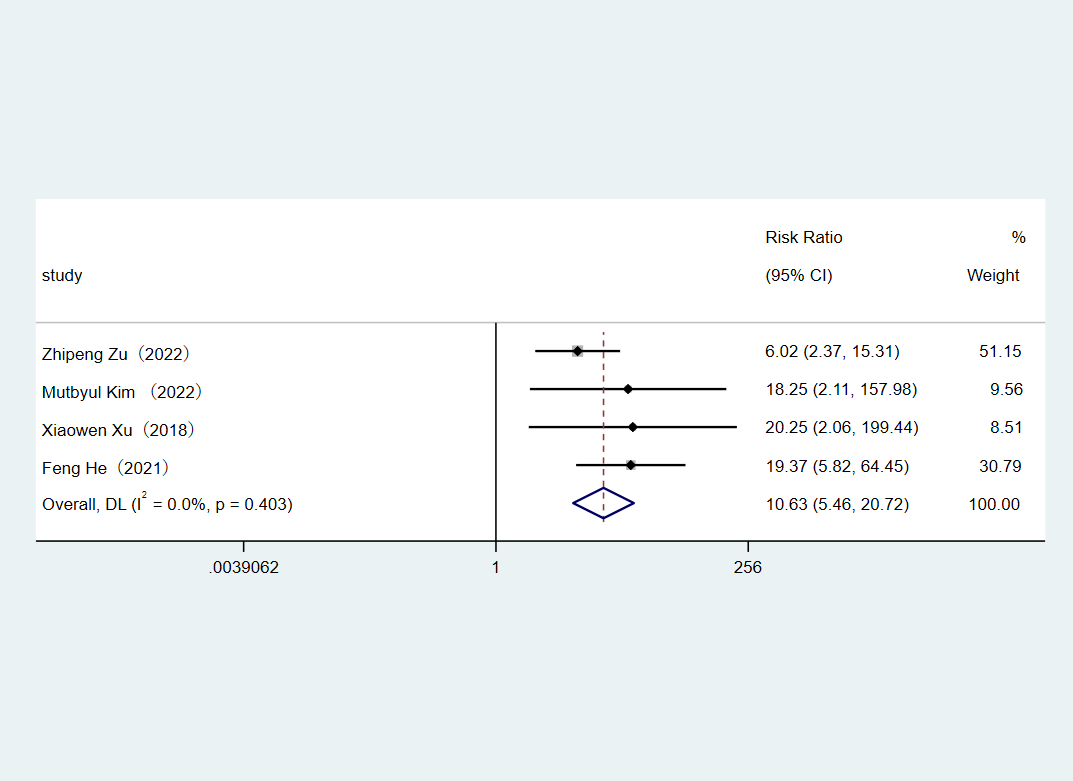


**Supplementary Figure 3.** Encephalopathy


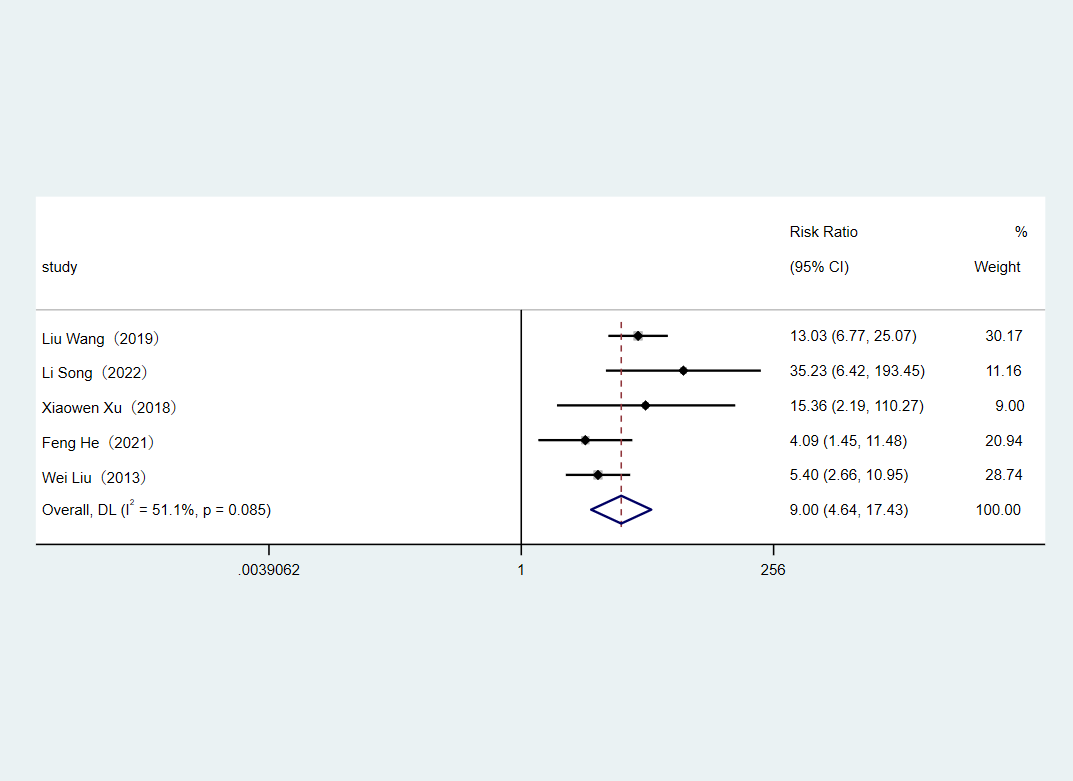


**Supplementary Figure 4.** The ratio of neutrophil to lymphocyte

A.


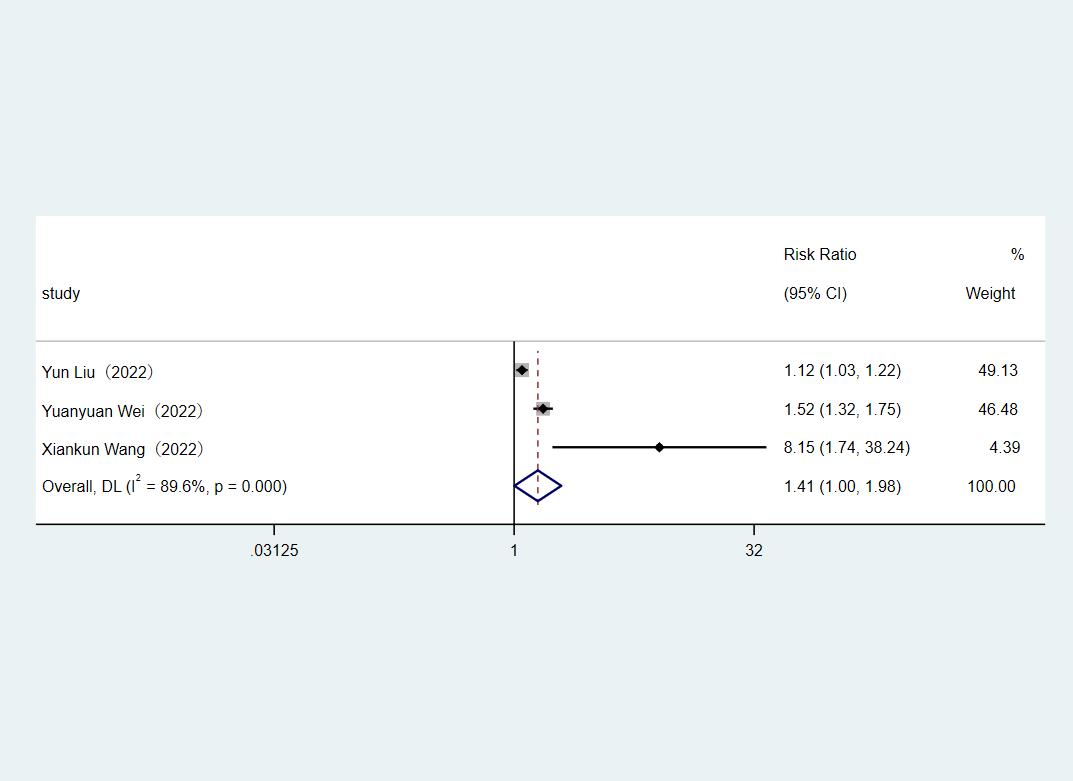


B


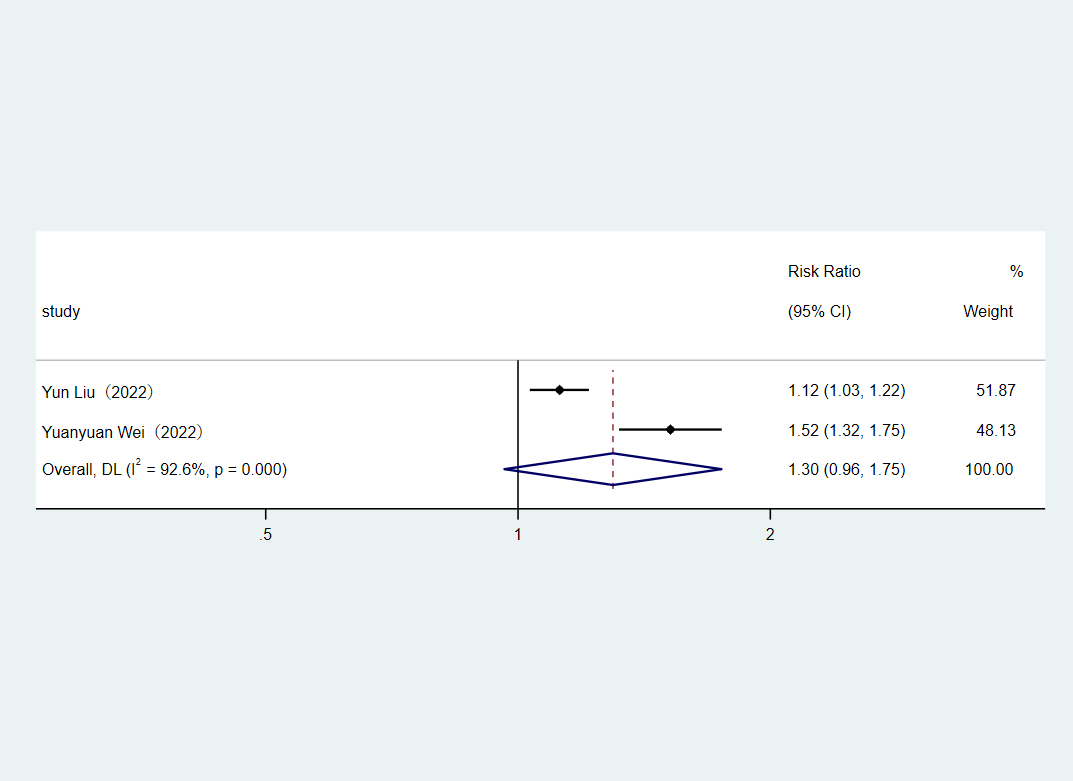


**Supplementary Figure 5.** Lymphocyte


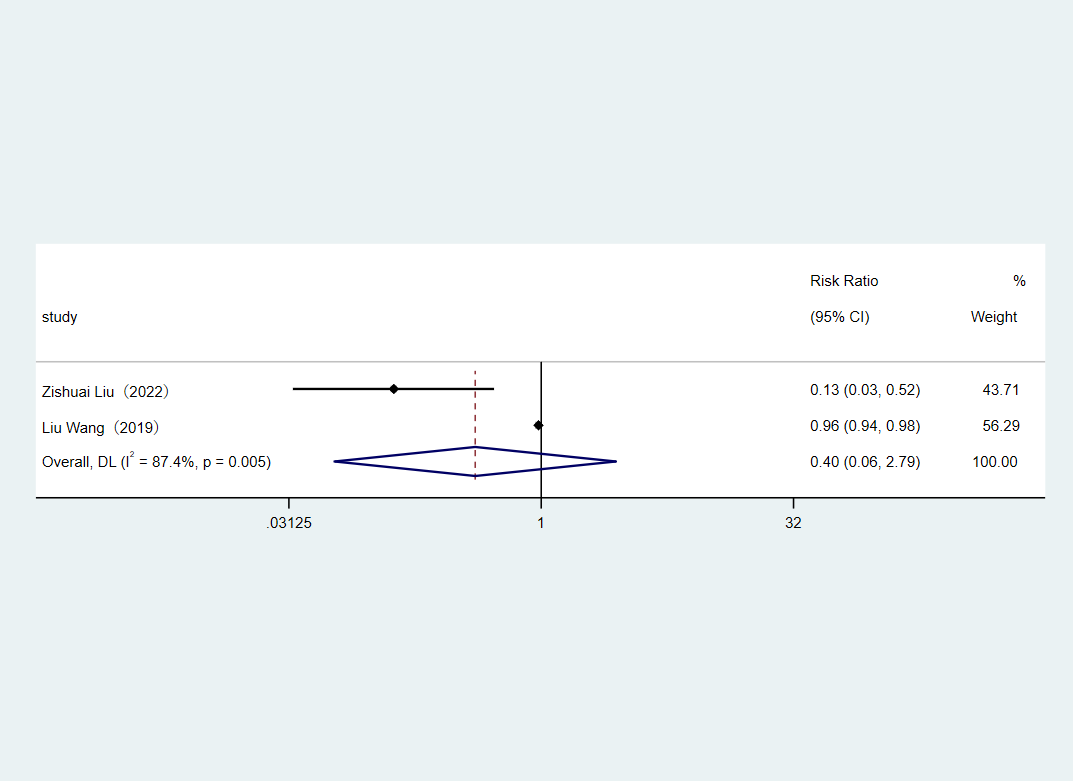


**Supplementary Figure 6.** The percentage of monocytes


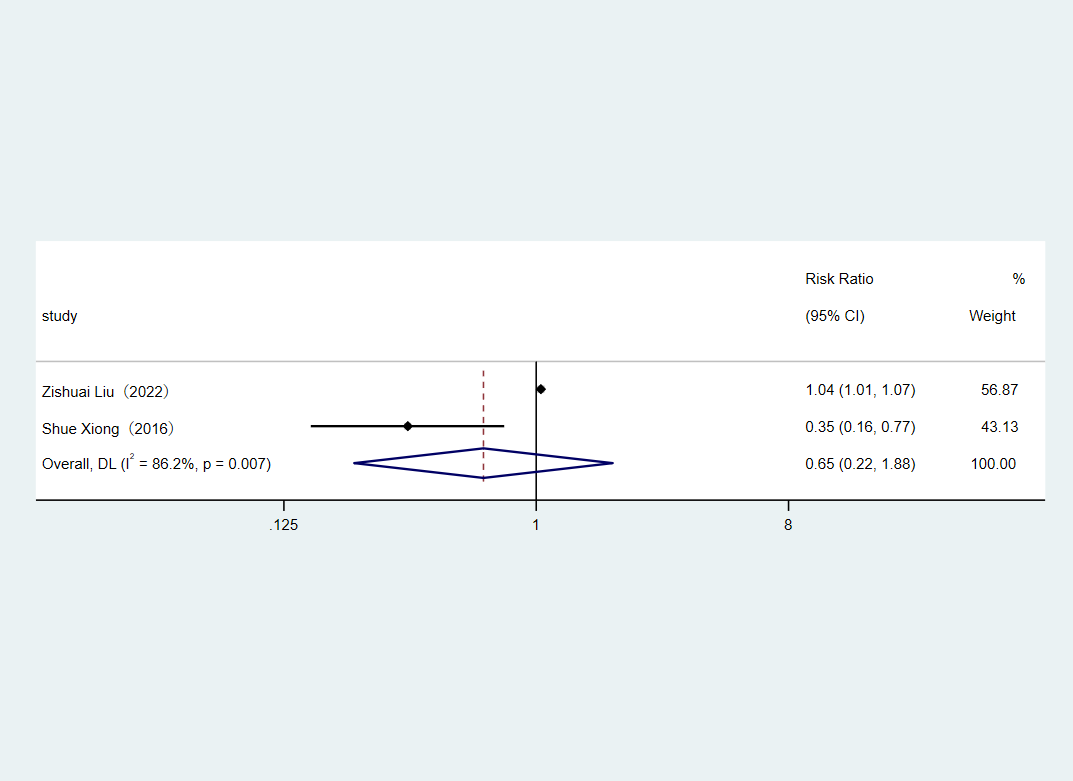


**Supplementary Figure 7.** AST

A


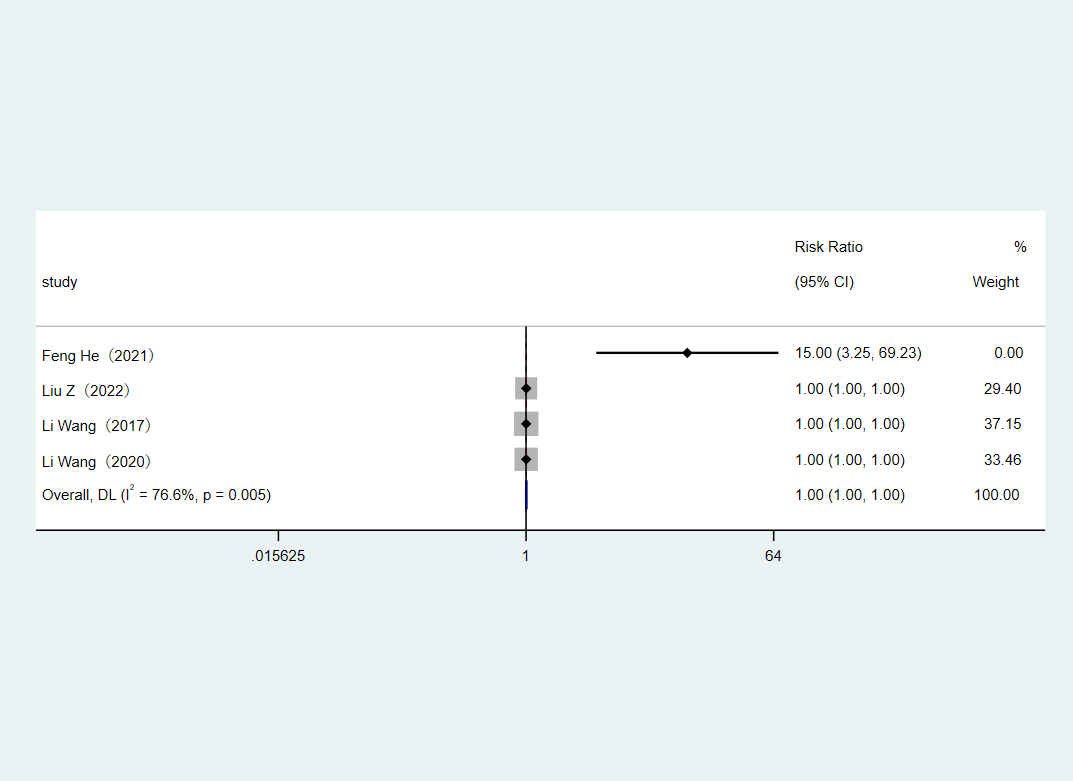


B


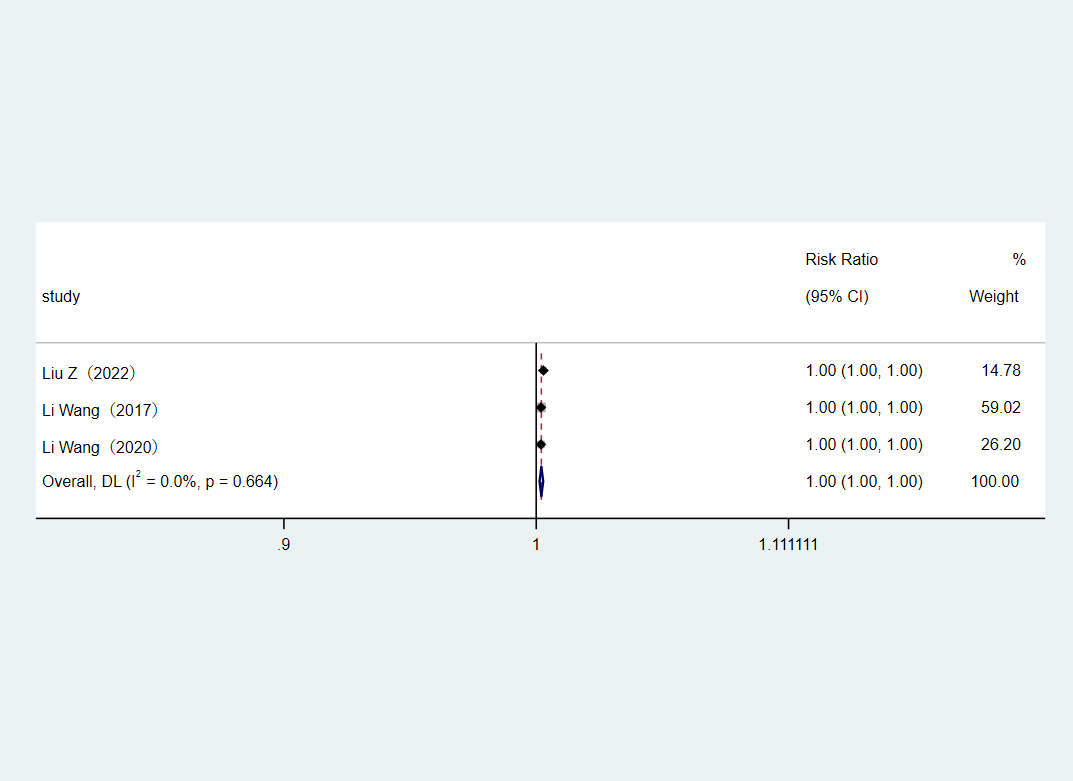


**Supplementary Figure 8.** Lactate dehydrogenase
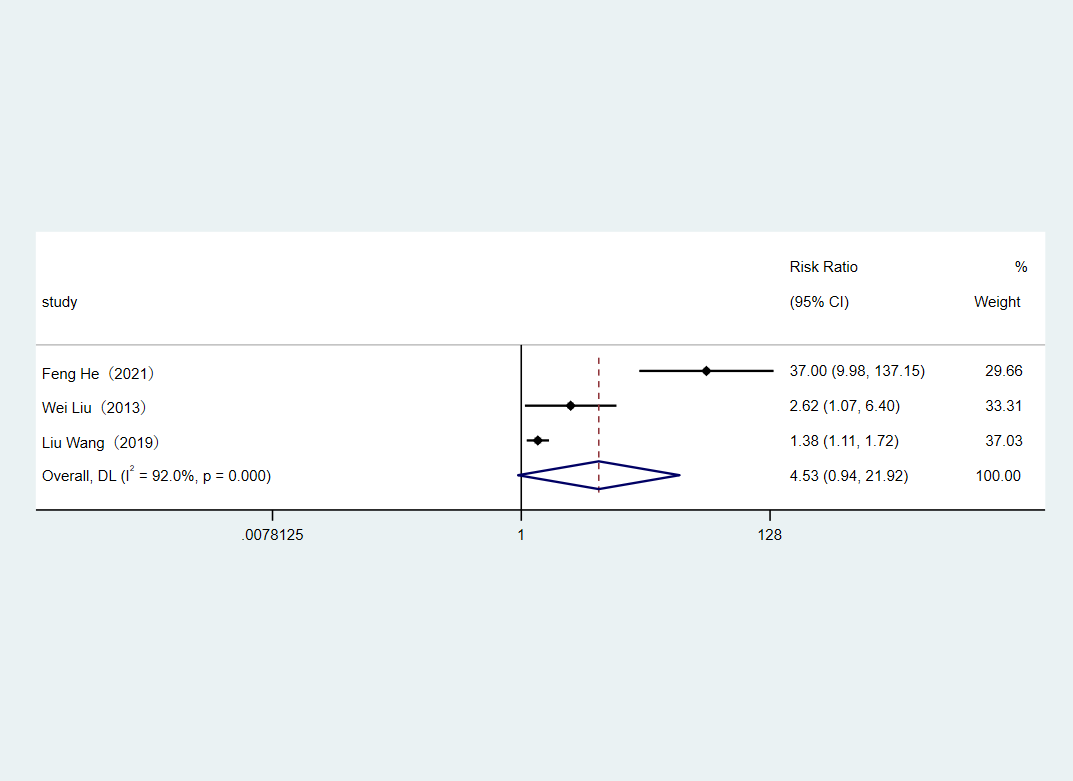


**Supplementary Figure 9.** AST/ALT-ratio


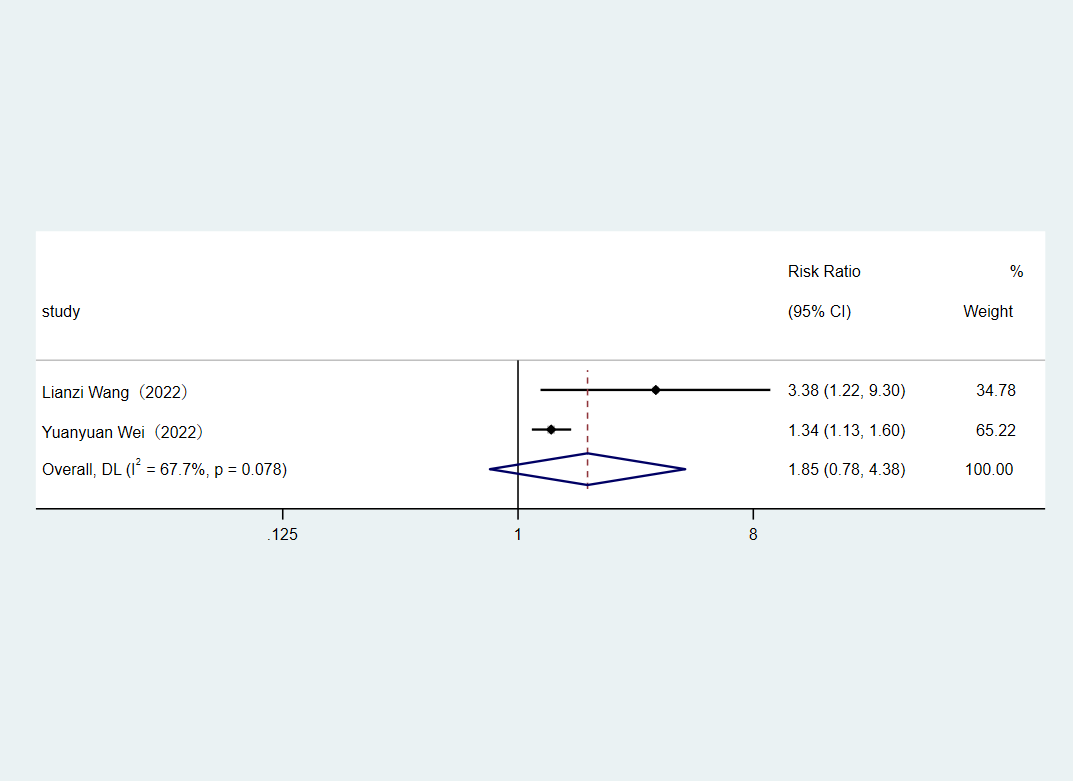


**Supplementary Figure 10.** Creatine kinase


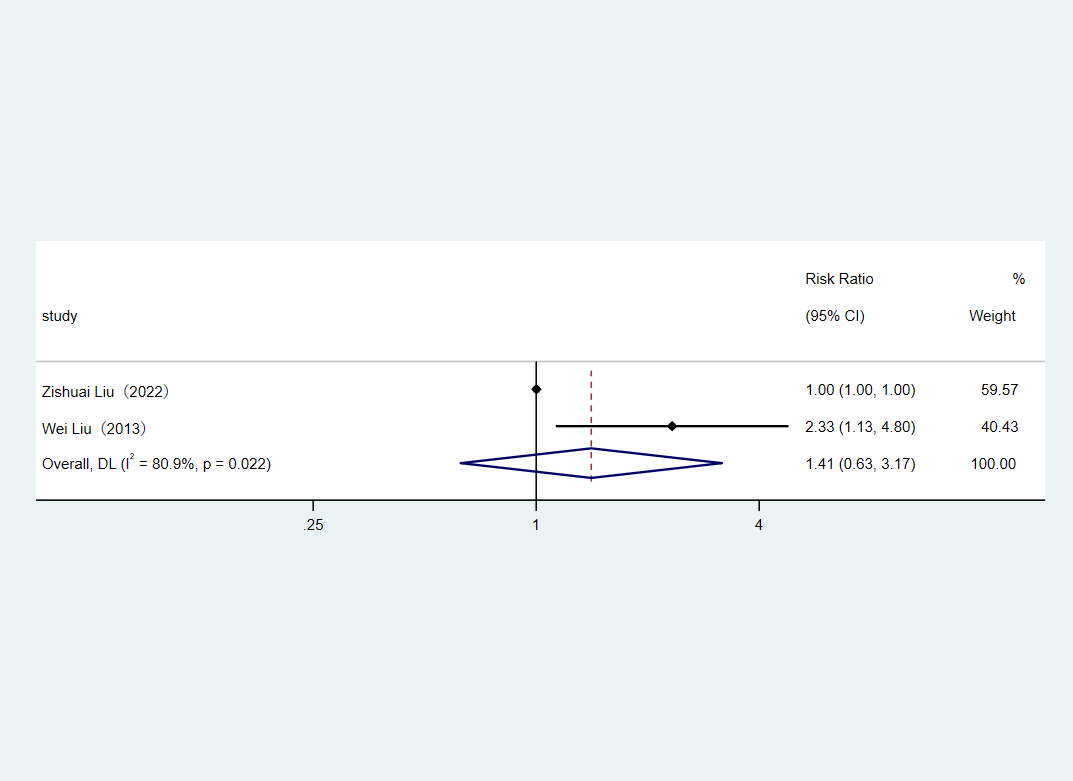


**Supplementary Figure 11.** The ratio of C-reactive protein to albumin;
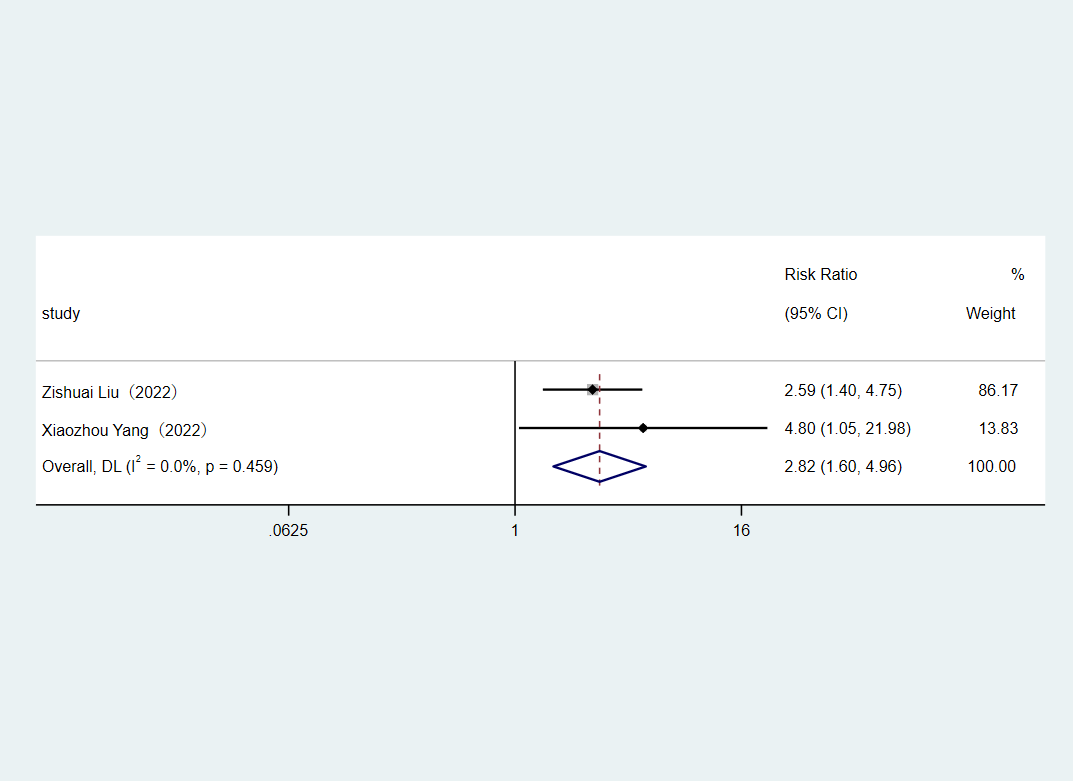


**Supplementary Figure 12.** Activated partial thromboplastin time


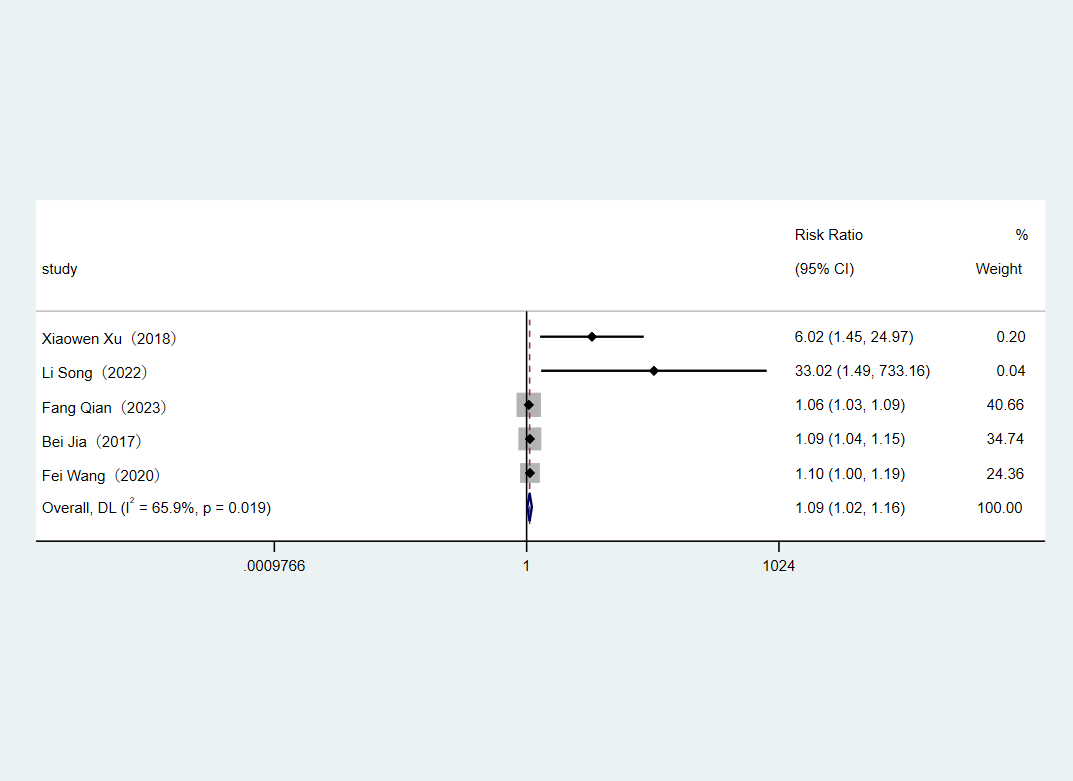


B


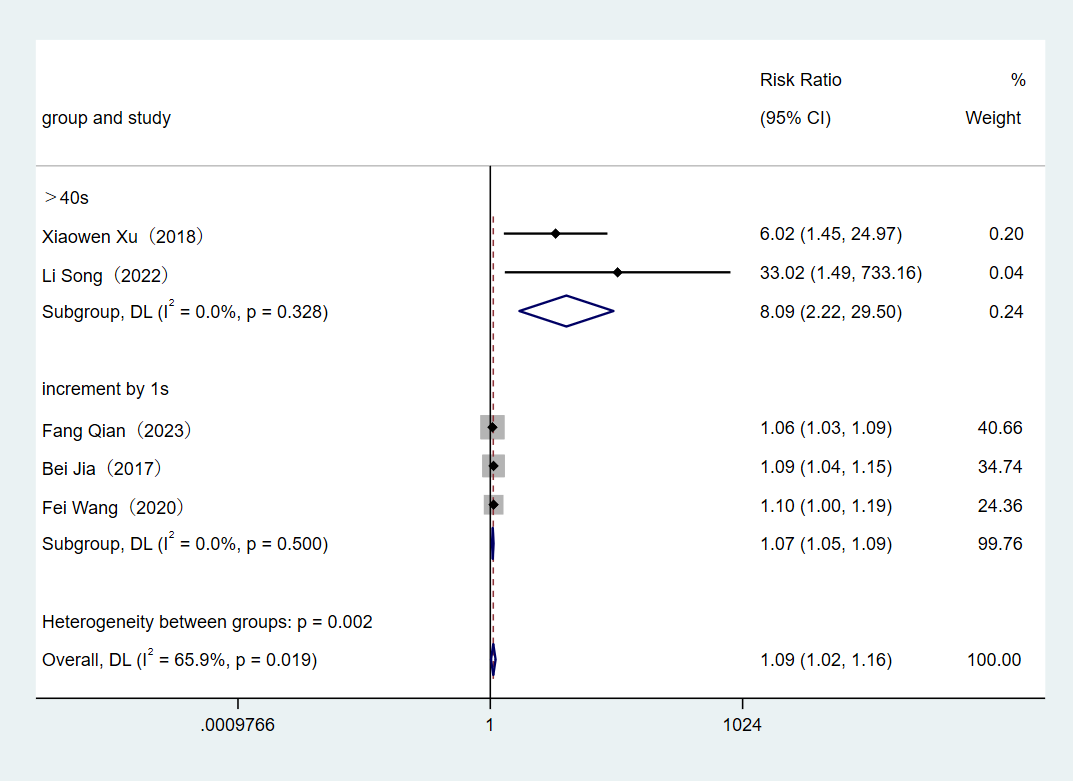


**Supplementary Figure 13.** Serum creatinine

A


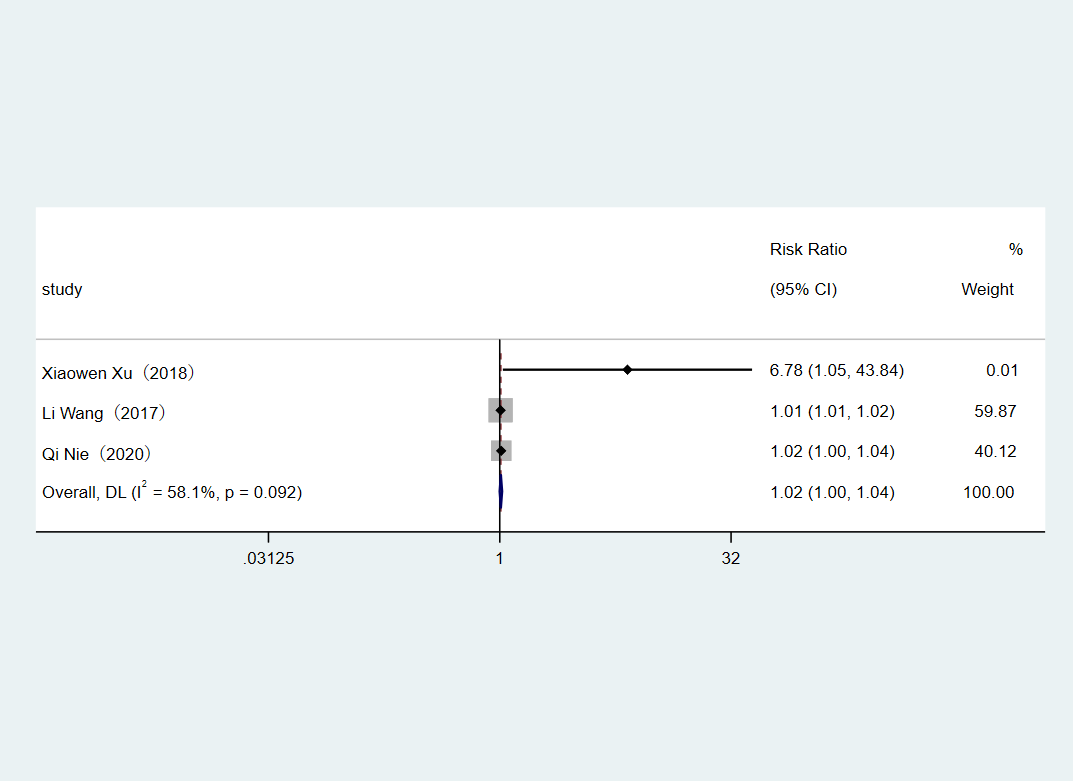


B


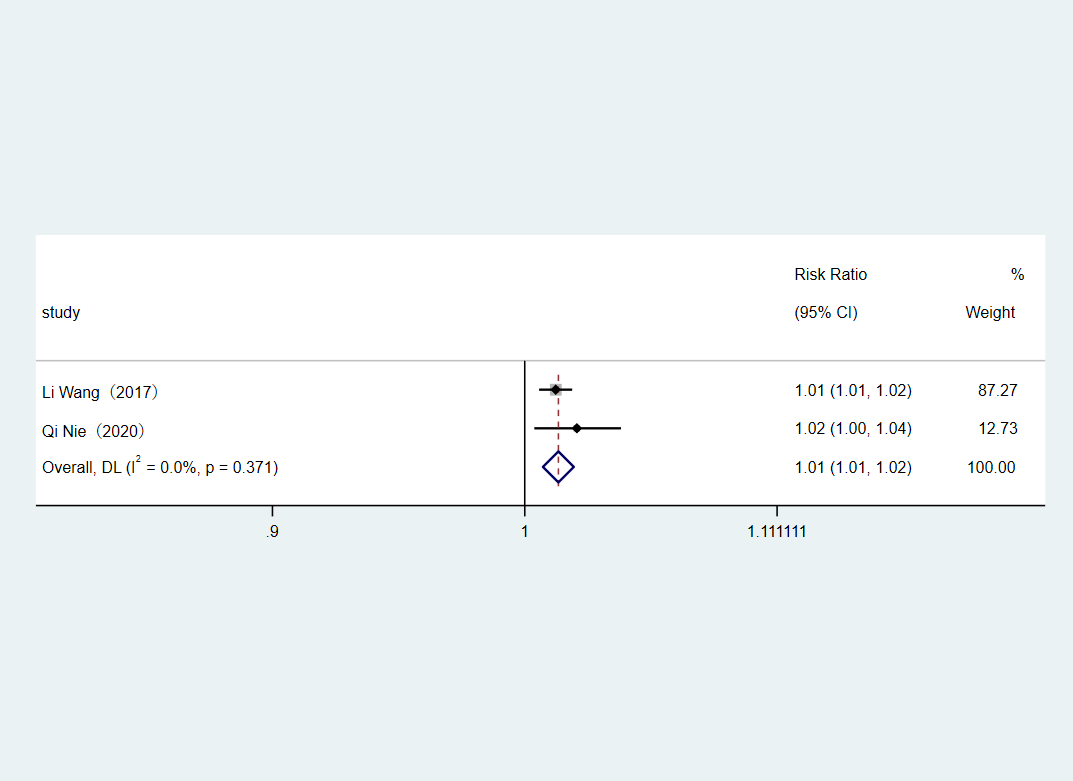


**Supplementary Figure 14.** Blood urea nitrogen

A


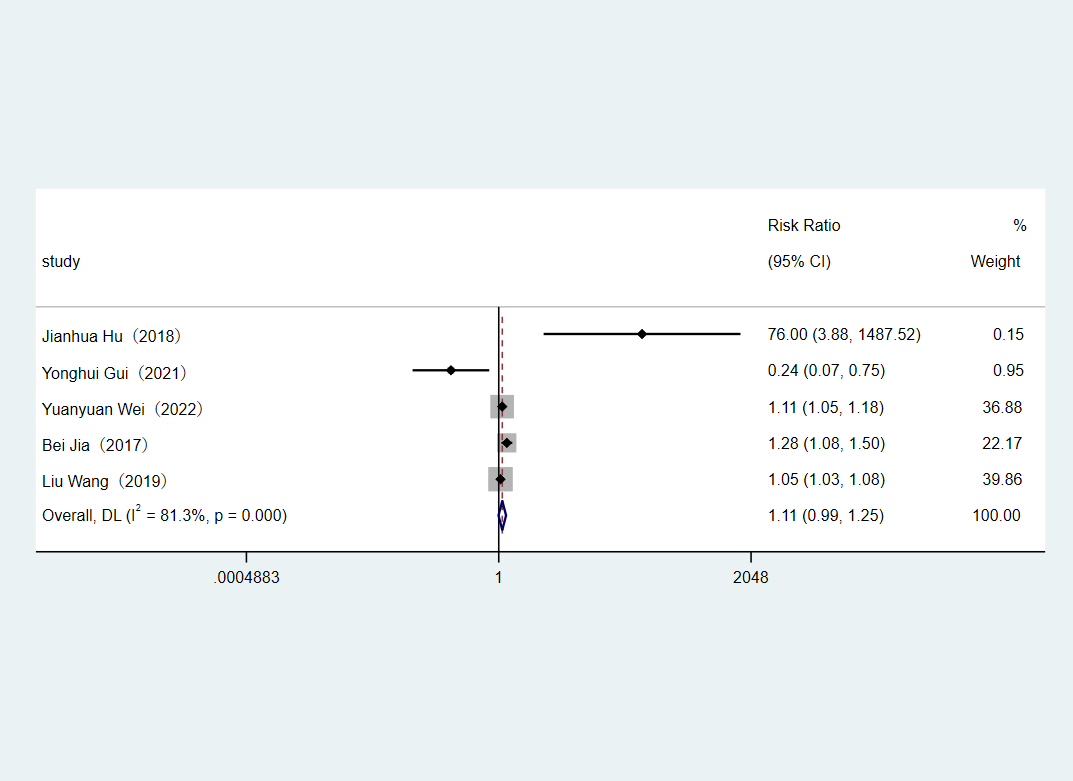


B


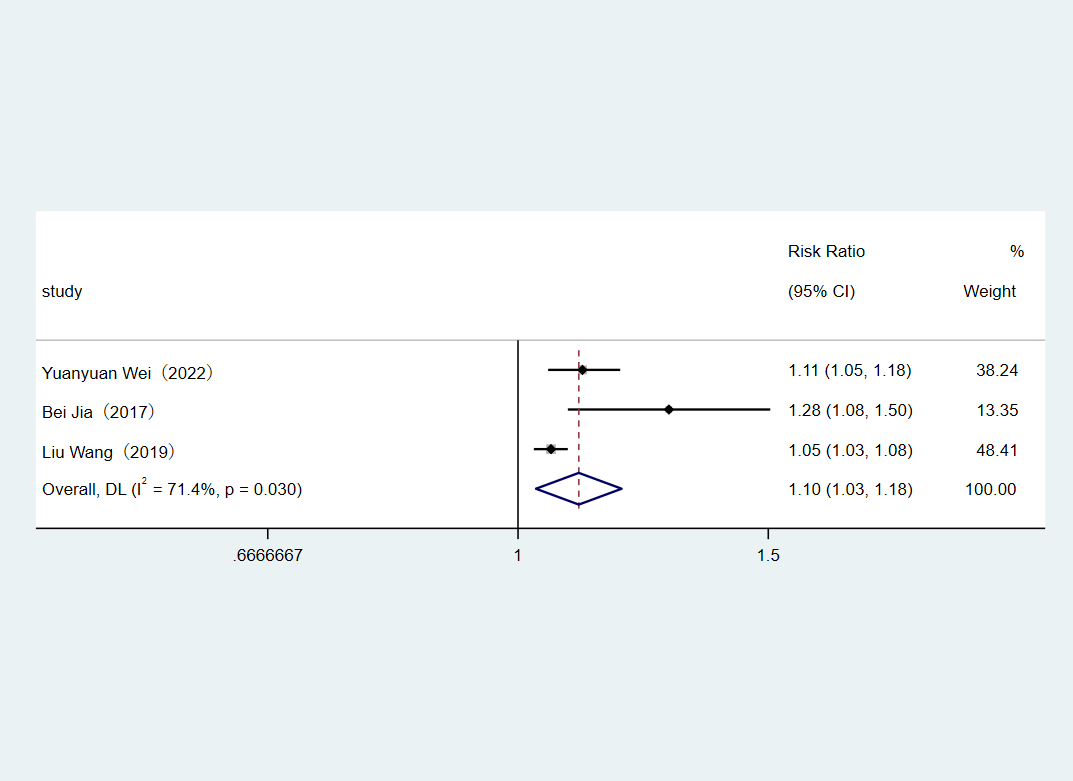


**Supplementary Figure 15.** Viral load

A


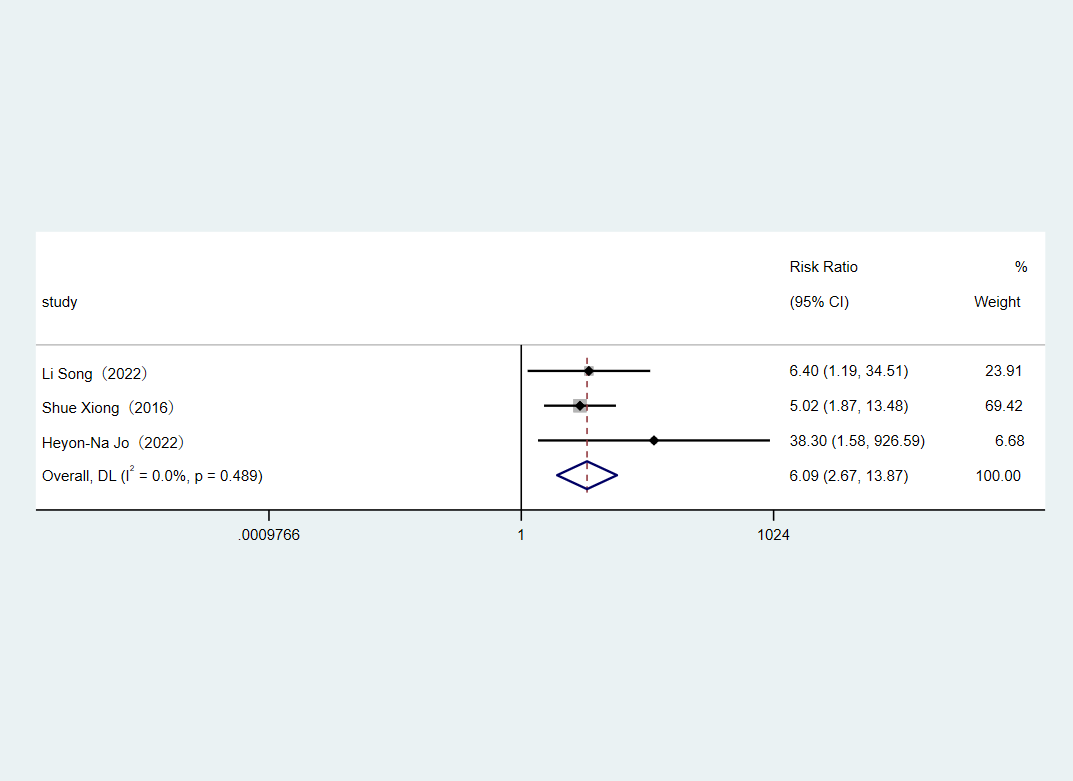


B


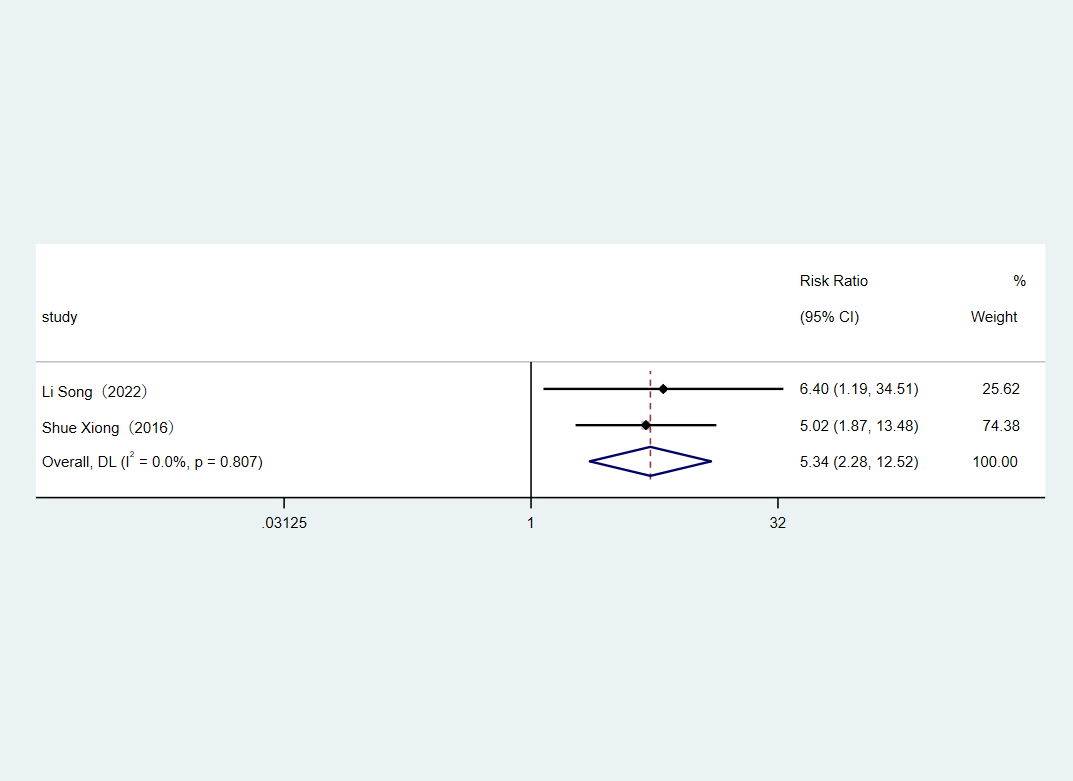


**Supplementary Figure 16.** Determination of prognostic risk factors in SFTS patients by the LASSO analysis.

**
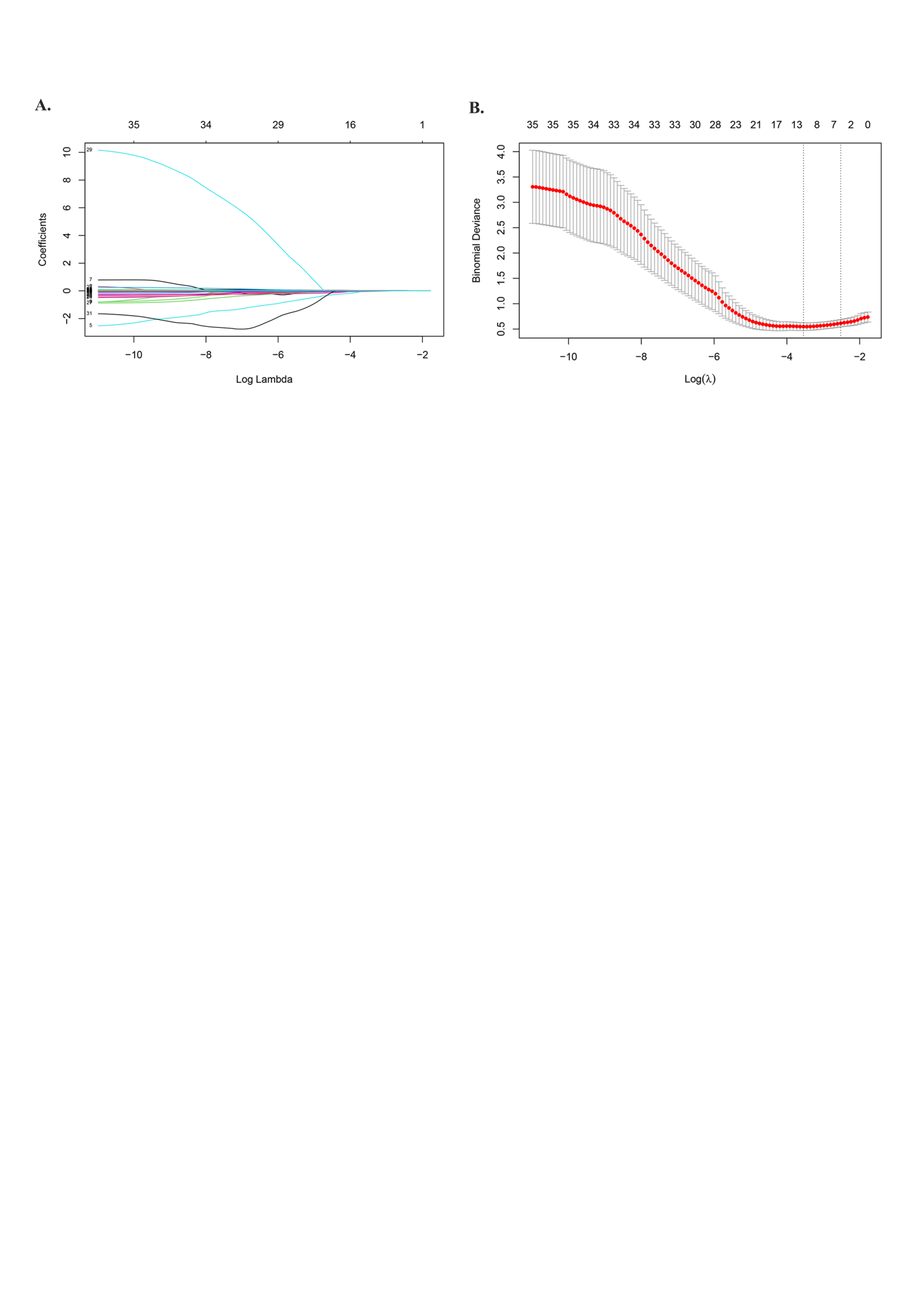
**
